# Usage, anti-inflammatory effect and safety of adjunctive acupuncture for cerebral infarction: an Apriori algorithm-based data mining and meta-analysis

**DOI:** 10.3389/fneur.2025.1546194

**Published:** 2025-05-19

**Authors:** Fangfei Yu, Shuang Li, Zunjiang Li, Zhaofan Mo, Rong Liu, Jiaying Zhao, Banghan Ding, Wei Yi, Nenggui Xu

**Affiliations:** ^1^Clinical Medical College of Acupuncture, Moxibustion and Rehabilitation, Guangzhou University of Traditional Chinese Medicine, Guangzhou, China; ^2^The First Clinical College of Guangzhou University of Chinese Medicine, Guangzhou, China; ^3^The Second Affiliated Hospital of Guangzhou University of Chinese Medicine, Guangzhou, China; ^4^Guangdong Provincial Hospital of Chinese Medicine, Guangzhou, China; ^5^The Second Clinical College of Guangzhou University of Chinese Medicine, Guangzhou, China; ^6^South China Research Center for Acupuncture and Moxibustion, Medical College of Acu-Moxi and Rehabilitation, Guangzhou University of Chinese Medicine, Guangzhou, China

**Keywords:** acupuncture, cerebral infarction, anti-inflammatory effect, meta-analysis, Apriori algorithm

## Abstract

**Background purpose:**

The adjunctive effect of acupuncture for cerebral infarction (CI) remains inconsistent. We aimed to determine its anti-inflammatory effect, assess safety, and summarize the adjunctive use of acupuncture for CI.

**Methods:**

We identified qualified randomized controlled trials (RCTs) from eight literature databases. Frequency analysis and Apriori association analysis were conducted using SPSS Modeler 18.0 and SPSS 26.0 software. A meta-analysis was performed using Stata 17.0 software. The credibility of the meta-results and the certainty of the evidence was assessed using trial sequential analysis (TSA) and GRADE methods, respectively.

**Results:**

A total of 43 RCTs were included, comprising 3,861 participants. Acupuncture with intermittent treatment (5–7 times per week), a combination of multiple points and multiple meridians (an average of 9.35 points in each prescription), typically lasting for 2–4 weeks, was commonly used for CI treatment. Meta-analysis indicated that the adjunctive use of acupuncture reduced levels of TNF-α (SMD = −1.36; 95% CI −1.51 to −1.20, *p* < 0.01), hs-CRP (SMD = −0.86; 95% CI −0.99 to −0.74, *p* < 0.01), and IL-6 (SMD = −0.85; 95% CI −1.08 to −0.62, *p* < 0.01), and decreased the rate of adverse events (RR = 0.71; 95% CI 0.49 to 1.01; *p* < 0.05). The certainty of the evidence was rated as moderate to high.

**Conclusion:**

Intermittent acupuncture treatment lasting at least 2 w was commonly used for CI patients, typically involving multiple acupuncture points and meridians. Acupuncture demonstrated an anti-inflammatory effect in the treatment of CI. However, due to the low quality of the existing literature, high-quality randomized controlled trials (RCTs) are required to confirm these results in the future.

**Systematic review registration:**

https://www.crd.york.ac.uk/prospero/, identifier CRD42017078583.

## Highlights

- It is first time to summarize the usage on the selection rules of acupuncture points for the treatment of cerebral infarction (CI).
- It is first time to determine the anti-inflammatory effect of adjunctive use of acupuncture for cerebral infarction (CI).
- Acupuncture is safe and effective to exert anti-inflammatory effect for CI.

## 1 Introduction

Cerebral infarction (CI) continues topose a significant health and socioeconomic issue, clinically manifesting as ischemic stroke. It occurs due to disruptions in cerebral blood flow, leading to damage to brain tissue and the death of neuronal cells (1, 2). Approximately 795,000 people experience new or recurrent strokes each year, with an average of one patient dying every 3.5 min due to stroke (3).

CI has been the leading cause of disability worldwide, which is mostly related to risk factors such as hypertension, diabetes mellitus, high cholesterol, and cardiovascular as well as cerebrovascular diseases (4, 5). Inflammation plays an important role in influencing the prognosis, pathophysiology, and pathology of ischemic infarction. For patients with CI, the activation of inflammation can aggravate blood–brain barrier (BBB) destruction, brain edema, and oxidative stress responses. It can also lead to intravascular obstruction and ischemic damage and is closely associated with a high rate of disability and an increased incidence of mortality (6–8). Currently, although anti-inflammation has been proven to benefit CI patients by preventing thrombosis and reducing bleeding risk, thereby improving prognosis and lowering the rate of adverse events (9, 10), effective methods to control or prevent inflammation in CI are still lacking (11, 12). Therefore, the development of new or alternative anti-inflammatory therapies for CI patients remains a significant challenge and an urgent need.

Acupuncture exerts its therapeutic effect by inserting sterile needles into the acupuncture points on the body's surface. It has been widely used for cerebrovascular issues in China due to its satisfactory clinical outcomes. Research suggests that acupuncture could promote neurogenesis and cell proliferation in the central nervous system, increase cerebral blood flow in ischemic areas, reduce apoptosis of nerve tissue in those regions, and improve memory after a stroke by enhancing long-term potentiation (LTP) in the hippocampal dentate gyrus (DG) and CA1 regions (13, 14). Importantly, acupuncture has been shown to regulate the expression of inflammatory cytokines, pro-inflammatory factors, and anti-inflammatory factors through multiple inflammatory signaling pathways, including tumor necrosis factor-α (TNF-α), interleukin-6 (IL-6), and interleukin-1β (IL-1β), which could significantly minimize the adverse effects on the brain and decrease recovery duration through its anti-inflammatory effects (15–17).

For primary outcomes, we hypothesize that acupuncture reduces levels of inflammatory markers such as TNF-α, IL-6, and hypersensitive C-reactive protein (hs-CRP), thereby improving the inflammatory status in CI patients. For secondary outcomes, we anticipate that acupuncture will increase the total clinical effective rate (TCER) and improve the National Institutes of Health Stroke Scale (NIHSS) score, reflecting enhanced clinical efficacy and neurological recovery. Additionally, we expect reductions in C-reactive protein (CRP) and IL-1β levels, further supporting the anti-inflammatory effects of acupuncture.

However, it still lacks a uniform usage or guidance of acupuncture for CI in practice, although a similar systematic review and meta-analysis have been performed to explore the therapeutic effect of acupuncture for CI (18–21). However, they mainly focus on the improvement of neurological function and clinical efficiency rather than on its anti-inflammatory effects and usage. Additionally, they lack subgroup analysis, sensitivity analysis, assessment of the quality of evidence, or trial sequential analysis (TSA), which reduces the robustness and confidence of their results. Thus, comprehensive and updated methods are still required to summarize the usage and evaluate the anti-inflammatory effects and safety of acupuncture for CI.

We have focused on collecting clinical evidence regarding the anti-inflammatory effects of acupuncture since 2017 (22). With an increasing number of relevant RCTs, we aim to demonstrate the clear anti-inflammatory effects of acupuncture and summarize its application for CI patients using the Apriori algorithm in data mining and meta-analysis. Our primary focus is on clarifying the following issues: (1) the anti-inflammatory effect of acupuncture for CI patients; (2) the safety of acupuncture for CI patients; (3) the frequency, duration, and application of acupuncture for CI, along with the criteria for selecting acupuncture points for CI treatment.

## 2 Data and methods

This systematic review and meta-analysis were conducted in accordance with the protocol published in 2019 (22) (PROSPERO registration No. CRD42017078583).

### 2.1 Database for search

Four English databases (PubMed, Cochrane Library, EMBASE, and the Web of Science) and four Chinese databases (China Science and Technology Journal Database, CBM, Wan-fang Database, and CNKI) were searched from January 1990 to May 2024 to identify qualified studies, regardless of language, age, race, country, or sex.

### 2.2 Criteria for studies included

#### 2.2.1 Type of participants

Patients diagnosed with CI (age > 18 years) were included, regardless of country, region, language, or gender.

#### 2.2.2 Type of interventions

**Acupuncture group:** Acupuncture treatments included all types of acupuncture therapy plus a control group, regardless of the type of acupuncture.

**Control group:** Conventional Western Treatment (CWT) for CI, which includes standard pharmacological and supportive treatments.

#### 2.2.3 Type of outcomes

**Primary outcomes:** The expected direction of the effect for primary outcomes includes the reduction of inflammatory markers such as TNF-α, IL-6, and hs-CRP, indicating an improved inflammatory status after treatment.

**Secondary outcomes:** We expected an increase in TCER and an improvement in the NIHSS score, indicating enhanced clinical efficacy and neurological recovery. In addition, we expected a reduction in CRP and IL-1β levels following treatment.

**Safety:** Adverse effect (AE) rates are defined as any negative acupuncture-related reactions occurring during or within 24 h after treatment, such as rash, gastrointestinal reactions, and dizziness. All adverse events were monitored and evaluated through clinical observation, patient self-report, and laboratory tests.

#### 2.2.4 Types of studies

Randomized controlled trials (RCTs).

### 2.3 Exclusion criteria

① Animal studies; ② review articles, commentaries, or editorials; ③ duplicate publications; ④ study protocols; ⑤ single-author articles; and ⑥ incomplete data.

### 2.4 Searching strategy

The MeSH terms of PICOS (Participants, Intervention, Comparison, Outcome, and Study design) were combined for a comprehensive literature search, including P+I+C+O+S, P+I+C+O, or P+I+O. P+I would be combined for searching when the number of included studies was too small. In such cases, we screened studies based on the inclusion and exclusion criteria. Key terms are provided in Supplementary material S1.

### 2.5 Data collection and analysis

#### 2.5.1 Selection of studies

Two reviewers screened RCTs independently by examining titles and abstracts before evaluating the full texts. Qualified studies were reviewed after removing duplicate literature. Each selected study met the inclusion and exclusion criteria. Any disagreements were resolved through discussion to achieve consensus among the reviewers.

#### 2.5.2 Data extraction and management

The details of the studies were screened independently by two reviewers and presented in a standardized table. Two authors extracted the data independently, including diagnostic criteria for CI diagnosis, study design, course of the disease, sample size, patient age, treatment details, acupuncture points, meridians associated with the acupuncture points, outcomes, and adverse events, among others.

#### 2.5.3 Evaluation of risk of bias

The risk of bias was assessed independently by two authors using the updated Cochrane Risk of Bias tool (RoB2), focusing on the randomization process, deviations from the intended intervention, missing outcomes, measurement of the outcome, and selection of reported results. Quality evaluation was categorized as “high risk,” “low risk,” or “some concerns.” Any discrepancies were resolved through discussion.

#### 2.5.4 Data synthesis and analysis

The frequency of acupuncture points, the meridians of acupoint selection, and the number of acupuncture points were calculated using IBM SPSS software (Version 22.0). An Apriori algorithm analysis was performed to explore the rules of acupuncture point selection using SPSS Modeler software (Version 18.0). Stata software (Version 17.0; MP) was used to pool the mean differences for continuous data, yielding standardized mean differences (SMD) and 95% confidence intervals (CI). For dichotomous data, relative risk (RR) and 95% CI were calculated. Heterogeneity between studies was presented as I^2^ values, classified as low, medium, and high for I^2^ values of 25%−50%, 50%−75%, and >75%, respectively (23, 24). Consequently, a random-effects model was selected for pooling when I^2^ > 75%. Sensitivity analysis, meta-regression analysis, and subgroup analysis were conducted to investigate sources of high heterogeneity. Otherwise, a fixed-effects model was selected for pooling (I^2^ ≤ 75%) (25, 26).

#### 2.5.5 Sensitivity analysis, meta-regression, and subgroup analysis

To enhance the robustness of the conclusions for the decision-making process, sensitivity analyses were conducted to examine significant heterogeneity among studies. We performed a sensitivity analysis to determine whether the heterogeneity significantly decreased after removing a single study. Subsequently, meta-regression analysis was employed (when the number of studies exceeded 10) to identify confounding factors that could contribute to high heterogeneity, and finally, subgroup analysis was performed based on these confounding factors.

### 2.6 Test sequential experiment

To illustrate and confirm the credibility of our results, we performed a sample size analysis using trial sequential analysis (TSA), which aims to eliminate the possibility of false positives. TSA was performed with 80% of the power and a 5% total risk of Type I error (27). Confidence intervals were adjusted for sparse data using TSA software (version 0.9.5.10 beta), and cumulative meta-analyses were retested. If the cumulative activity Z-curve crosses the TSA monitoring boundary or the required information size (RIS) line, we can conclude that reliable conclusions can be drawn without further research.

### 2.7 Evidence confidence

The certainty of evidence was assessed using the GRADE (Grading of Recommendations, Assessment, Development, and Evaluation) methodology according to the instructions provided on the website (https://www.gradepro.org/) (28). Initially, RCT evidence was classified as high quality, but it may be downgraded due to risks such as bias, inaccuracy, inconsistency, informal practices, or publication bias, among others. Finally, the quality of the evidence was divided into four levels: “high,” “moderate,” “low,” and “very low.”

## 3 Results

### 3.1 Results of the RCT selection process

**Figure 1:**
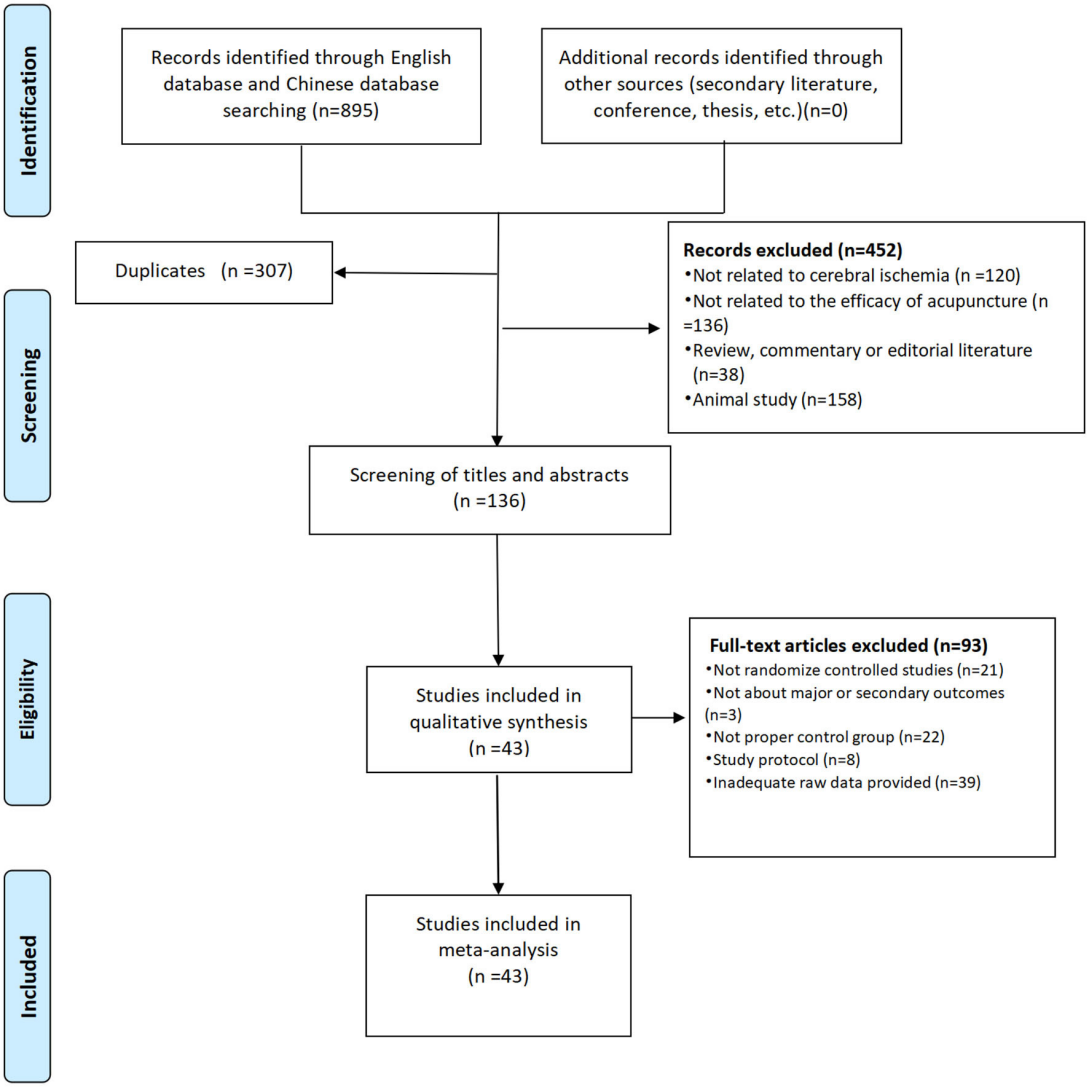
PRISMA flow diagram illustrating the literature screening and selection process.

Our initial search identified 895 records, from which 307 articles were removed after eliminating duplicates. Additionally, 452 records comprised of reviews, commentaries, editorial literature, animal studies, and unrelated studies were removed based on title and abstract evaluations. Upon reviewing the full-text articles, 93 records including non-RCT studies, research protocols, and articles with inappropriate control groups, incomplete original data, single authors, or inappropriate primary or secondary results were eliminated. Finally, 43 RCTs were included, involving a total of 3,861 participants (29–71). Table 1 presents the characteristics of the included RCTs, while Figure 1 details the PRISMA flowchart for this review.

**Table 1:** The characteristics of the included RCTs.

**Study**	**Study design**	**Diagnosis criterion**	**The course of the disease**		**Sample size**		**Age**		**Intervention treatment**		**Usage**	**Needle retention time**	**Period of treatment (d)**	**Outcome**
			**C**	**E**	**C**	**E**	**C**	**E**	**Control**	**Acupuncture**				
Zhao et al. (29)	RTN	o	6.50 ± 1.25 h	6.45 ± 1.22 h	38	38	62.55 ± 10.18	62.80 ± 10.22	CWT	CWT+electroacupuncture	1 time/d	30 min	14	①II④II⑥⑦
He et al. (30)	RTN	g	10.04 ± 2.79w	9.96 ± 2.53w	50	50	59.31 ± 6.28	58.79 ± 5.10	Rosuvastatin Calcium Tablets	Rosuvastatin Calcium Tablets+Warm acupuncture	1 time/d, 5 times a week	30 min	28	①II②III⑥
Lv et al. (31)	RTN	NR	11.52 ± 3.23 d	11.38 ± 3.18d	48	52	62.15 ± 4.33	61.86 ± 4.45	aspirin+RRT	aspirin+RRT+acupuncture	NR	30 min	2 m	①II⑤⑧
Li et al. (32)	Random	NR	3.28 ± 0.44w	3.25 ± 0.39w	32	33	54.98 ± 7.74	55.01 ± 7.78	RRT	RRT+acupuncture	1 time a week	30 min	12 w	①II②III③V⑦
Li et al. (35)	Random, class stratum sampling	g	3.15 ± 0.49h	3.12 ± 0.45h	54	54	56.29 ± 6.40	56.25 ± 6.32	CWT+Citicoline and alteplase	CWT+Citicoline and alteplase+acupuncture	1 time/d, 6 time a week	20 min	28	①III②III③III⑤
Zhang et al. (36)	RTN	g	3.56 ± 0.62h	3.63 ± 0.57h	34	34	59 ± 8	59 ± 8	Interventional Thrombolysis in Cerebral Artery+hyperbaric	Interventional Thrombolysis in Cerebral Artery+hyperbaric+acupuncture	1 time/d	20 min	14	①II④II⑤⑦⑧
Sun et al. (37)	RTN	o	17.32± 2.65h	17.37± 2.64h	51	51	57.86 ± 4.25	57.81 ± 4.29	CWT (Butylphthalide)	CWT (Butylphthalide) +acupuncture	1 time/d	30 min	28	①II⑤⑧
Wang et al. (39)	RTN	b	7.1 ± 1.5w	6.9 ± 1.4w	75	75	66.8 ± 8.0	67.0 ± 6.9	CWT+RRT	CWT+RRT+acupuncture	1 time/d, 5 times a week	60 min	8 w	①I⑥
Zhao et al. (40)	Random	g	17.567 ± 4.191d	17.800 ± 3.906d	30	30	63.933 ± 30.095	63.733 ± 2.572	RRT	RRT+acupunture abdominal	1 time/d, 5 time a week	30 min	14	①NR
Xia et al. (41)	RTN	b	6.13 ± 1.45w	6.50 ± 1.73w	45	45	57.18 ± 6.48	56.61 ± 6.29	CWT	CWT+Warm acupuncture	1 time/d, 5 times a week	30 min	28	④II⑤⑦⑧
Li and Qing (43)	RTN	o	7.31 ± 0.58d	7.28 ± 0.64d	62	62	64.48 ± 5.62	65.17 ± 5.76	CWT	CWT+acupuncture	1 time/2 d	30 min	30	①III②II⑤
Hua et al. (45)	RTN	g	NR	NR	50	50	62 ± 12	64 ± 9	CWT	CWT+acupuncture	1 time/d	30 min	14	②III④II⑤⑧
Guo and Kou (46)	RTN	b	NR	NR	50	50	NR	NR	CWT	CWT+acupuncture	1 time/d	NR	28	①III②II③IV
Wu et al. (47)	RTN	g	NR	NR	31	32	66.37+ 3.62	65.92 ± 3.79	CWT+RRT	CWT+RRT+acupuncture	1 time/d	40 min (20 am and 20 pm)	28	①IV②II⑤⑥⑦
Zhu et al. (48)	RTN	d	NR	NR	48	52	71 ± 2	71 ± 2	CWT+Edaravone injection	CWT+Edaravone injection+Warm acupuncture	1 time/d	30 min	14	①II②II③II⑤⑦⑧
Zhao et al. (51)	RTN	g	7 ~ 14 d		27	27	NR	NR	RRT	RRT+acupuncture	5–6 times a week	7–8h	14	①III
Cai et al. (55)	RTN	d	2.85 ± 1.26d	2.73 ± 1.30d	83	83	63.09 ± 8.84	62.92 ± 9.71	CWT+RRT	CWT+RRT+electronacupuncture	1 time/d	30 min	28	①II②II③III⑦
Zhang et al. (57)	RTN	d	NR	NR	19	18	66.53 ± 8.79	64.89± 9.42	CWT	CWT+acupuncture	1 time/d, 6 times a week	30 min	28	②I⑤⑥⑦
Ruan and Zhang (67)	Random	d	NR	NR	20	20	60.11 ± 8.01	62.20 ± 9.05	CWT	CWT+scalpel	1 time/d	20 min	14	②II⑤
Mu et al. (71)	Random, single-blind	d	2.18 ± 0.96d	2.11 ± 1.09d	30	30	66.5 ± 8.81	69.5 ± 7.10	CWT	CWT+scalpel	NR	No needle retained	20	②II
Wang et al. (76)	Stratified block randomization	d	13.7 ± 7.7h	13.0 ± 7.7h	30	31	55.8 ± 9.9	54.9 ± 10.7	CWT	CWT+head acupuncture	1 time/d	30 min	7	①III②III④II⑤⑥
Xi et al. (34)	RTN	g	≤30 d		48	48	45~75		CWT+RRT	CWT+RRT+acupuncture	1 time/d, 5 times a week	30 min	8w	①II②II⑦
Xu et al. (38)	RTN	NR	10.65 ± 2.57d	11.26 ± 2.29d	34	34	59.65 ± 9.90	59.68 ± 7.81	CWT+Hyperbaric oxygen therapy	CWT+Hyperbaric oxygen therapy+acupuncture	1 time/d	90 min	10	⑥⑦⑧
Shan et al. (42)	RTN	g	<48 h		31	31	61.85 ± 6.96	60.99 ± 6.79	CWT	CWT+acupuncture	1 time/d	No needle retained	21	⑤⑥
Jiang et al. (44)	RTN	o	NR	NR	20	20	72.35 ± 13.90	68.55 ± 15.68	CWT	CWT+acupuncture	1 time/d	No needle retained	7	③II
Zhang et al. (49)	Random	d	16.21 ± 2.12y	15.39 ± 2.73y	50	50	65.23 ± 10.75	64.50 ± 7.42	CWT	CWT+acupuncture	NR	30 min	28	②II⑤
Xue et al. (50)	RTN	o	8.12 ± 0.39h	8.01 ± 0.43h	40	40	59.26 ± 7.63	60.14 ± 7.82	CWT	CWT+acupuncture	1 time a week	30 min	28	①II④II⑤⑦
Tao et al. (52)	RTN	b	17.24 ± 8.23 h	17.87 ± 9.05h	71	71	65.03 ± 6.82	64.67 ± 6.31	CWT	CWT+acupuncture	1 time/d	No needle retained	14	⑤⑥⑦
Guo and Wang (53)	RTN	d	<24h		52	52	60.97 ± 1.98	61.05 ± 2.15	CWT	CWT+acupuncture	1 time/d	30 min	7	⑥⑦
Xiang et al. (54)	RTN	g	NR	NR	60	60	55.06 ±0.35	55.04 ± 0.34	CWT+RRT	CWT+RRT+acupuncture	1 time/d, 5 times a week	30 min	14	③II⑤⑦
Wang et al. (56)	Random	d	33.82 ± 3.97h	34.02 ± 4.02h	32	32	56 ± 7	56 ± 7	Edaravone and plasmin injection	Edaravone and plasmin injection +acupuncture	1 time/d, 5 times a week	No needle retained	14	③II⑤⑧
Li et al. (58)	Random, toss a coin	g	6.03 ± 1.21h	5.94 ± 1.27h	130	130	62.72 ± 5.84	62.82 ± 5.75	CWT	CWT+acupuncture	1 time/d	30 min	14	①II②II⑥⑦
Liu and Li (60)	RTN	d	32.23± 12.57h	31 ± 11.32 h	33	33	55.45 ± 6.03	54.15 ± 6.87	Edaravone and plasmin injection	Edaravone and plasmin injection+acupuncture	1 time/d, 5 times a week	30 min	14	⑧
Wang et al. (61)	Random	d	8.98 ± 5.39d	10.52 ± 4.74d	30	30	57 ± 6	53 ± 8	RRT	RRT+acupuncture	1 time/d	45 min	10	⑥
Sun et al. (62)	RTN	NR	NR	NR	51	51	58.63 ±8.67	57.67 ±9.21	CWT	CWT+acupuncture	1 time/d	50 min	28	⑤
Yu and Wang (63)	Random	d	2.3 ± 1.1 month	2.1 ± 0.9 month	30	30	60.1 ± 3.5	59.6 ± 2.9	RRT	RRT+acupuncture	1 time/d	30 min	20	③II
Wang et al. (64)	RTN	d	<7 d		40	40	60 ± 8	60 ± 9	CWT	CWT+acupuncture	1 time/d	60 min	14	⑥⑦
Li et al. (65)	Random	d	NR	NR	30	30	62 ± 11	62 ± 11	CWT	CWT+acupuncture	1 time/d	6 h	14	⑥
Li et al. (32)	Random	NR	1.45 ± 0.55 d	1.53 ± 0.47 d	50	50	59.75 ±4.25	58.65 ±4.35	CWT	CWT+acupuncture	1 time/d	30 min	14	⑤⑥⑦⑧
Sang et al. (66)	RTN	d	NR	NR	40	40	59.65 ± 8.34		CWT	CWT+acupuncture	1 time/d	30 min	14	⑤
Guo et al. (68)	RTN	d	<3 d		33	34	41 73	42 75	RRT	RRT+acupuncture	1 time/d	30 min	14	⑥⑦⑧
Wang et al. (69)	RTN	d	7.1± 2.3 h	7.1± 2.8 h	73	73	54.7 ± 10.1	55.1 ± 12.3	CWT	CWT+acupuncture	1 time/d, 5 times a week	30 min	21	⑥
Sang et al. (70)	RTN	NR	NR	NR	40	40	NR	NR	CWT	CWT+acupuncture	NR	NR	14	①VI②III

E, acupuncture group; C, control group; RTN, random number table method; ACI, acute cerebral infarction; CI, cerebral infarction; CWT, conventional western treatment; RRT, regular rehabilitation training; d, diagnosis of various types of cerebrovascular diseases (1995, 1996, or 1998 version) published at The Fourth China Academic Conference on Cerebrovascular Diseases; g, guidelines for the diagnosis and treatment of acute ischemic stroke in China (2010, 2013, 2014, 2017, or 2018 version); o, other lectures unrelated to CI diagnosis; b, based on laboratory examination, symptoms, and signs of CI; NR, not reported; ① tumor necrosis factor-α(TNF-α); ② interleukin-6 (IL-6); ③ interleukin−1β(IL−1β); ④ C-reactive protein (CRP); ⑤ total clinical effective rate(TCER); ⑥ hypersensitive-c-reactive-protein (hs-CRP); ⑦ NIHSS score; ⑧ adverse event(AE) rate. The unit of outcome measure: I. pg/L; II. pg/ml or ng/L; III. ng/ml or μg/L; IV. μg/ml or mg/L; V. mg/ml; VI. fmol/L.

### 3.2 Systematic review

#### 3.2.1 Characteristics of the included RCTs

A total of 43 studies with 3,861 patients with CI were published from 2008 to 2022, including 1,925 in the control group and 1,936 in the acupuncture group. Among these, TNF-α, IL-6, and hs-CRP were regarded as outcomes in 18 articles (29–31, 33–37, 39, 40, 43, 46–48, 50, 51, 55, 59), 17 articles (30, 33–35, 43, 45–49, 55, 57–59, 67, 70, 71), and 17 articles (29, 30, 32, 38, 39, 42, 47, 52, 53, 57–59, 61, 64, 65, 68, 69), respectively. Nine articles used CRP as the outcome indicator (33, 35, 44, 46, 48, 54–56, 63), and six articles used IL-1β as the outcome indicator (29, 36, 41, 45, 50, 59). TCER and NIHSS scores were used as indicators in 21 articles (31, 32, 35–37, 41–43, 45, 47–50, 52, 54, 56, 57, 59, 62, 66, 67) and 18 articles (29, 32–34, 36, 38, 41, 47, 48, 50, 52–55, 57, 58, 63, 68), respectively. Additionally, only 11 studies reported the AE rate (31, 32, 36–38, 41, 45, 48, 56, 60, 68).

The majority of studies (30/out of 43, 69.8%) used the random table number method for allocation (29–31, 34, 36–39, 41–48, 50–55, 57, 60, 62, 64, 66, 68–70), 10 studies lacked details about the random allocation methods (32, 33, 40, 49, 56, 61, 63, 65, 67, 71), and three studies used other random allocation methods (35, 58, 59).

Apart from one study, the majority of studies (42/43, 97.7%) did not report the detailed blinding process (29–70). The sample size of the majority of studies (35/43, 81.4%) ranged from 60 to 160 (29–32, 34–43, 45–50, 52, 53, 56, 59–66, 68–71), four studies had fewer than 60 (44, 51, 57, 67), and two studies had more than 160 (55, 58). Regarding diagnostic criteria, 11 studies were consistent with the Guidelines for the Diagnosis and Treatment of Ischemic Stroke in China (2010, 2013, 2014, 2017, or 2018 version) (30, 34–36, 40, 42, 45, 47, 51, 54, 58). A total of 17 studies followed the diagnostic criteria outlined in the Fourth China Academic Conference on Cerebrovascular Diseases (1995, 1996, or 1998 versions) (48, 49, 53, 55–57, 59–61, 63–69, 71), five studies used other academic references related to CI diagnosis (29, 37, 43, 44, 50), and four studies based diagnosis on laboratory tests, clinical symptoms, and signs of CI (39, 41, 46, 52). Six studies did not report the diagnostic criteria used (31–33, 38, 62, 70). Thirteen studies included patients within 48 h of onset (29, 35–37, 42, 50, 52, 53, 56, 58–60, 69), 12 studies included patients within 2 d to 30 d (31–34, 38, 40, 51, 55, 61, 64, 68, 71), and five studies included patients with disease duration longer than 1 month (30, 39, 41, 49, 63), but 12 studies did not specify the disease duration (44–48, 54, 57, 62, 65–67, 70). Finally, the average age of the patients with CI ranged from 45 to 75 in the majority of studies (40/43, 93.0%) (29–45, 47–50, 52–69, 71), while three studies did not report patient age (46, 51, 70).

#### 3.2.2 Usage of acupuncture

In terms of acupuncture usage, the majority of studies (35 out of 43, 81.4%) reported that participants received acupuncture treatment 5 to 7 times a week (29, 30, 32, 34–42, 44–48, 51–69), and the duration of acupuncture treatment in 25 studies (25/out of 43, 58.2%) was 30 min (29–34, 37, 40, 41, 43, 45, 48–50, 53–55, 57–60, 63, 66, 68, 69). Additionally, in the majority of studies (33/43, 76.7%), the duration ranged from 14 d to 28 d (29, 30, 32, 34–37, 39–42, 45–52, 54–58, 60, 62–71), except for 10 studies [five studies lasted more than 28d (31, 33, 34, 39, 43), three studies were 7d (44, 53, 59), and two studies were 10d (38, 61)]. Regarding the types of acupuncture, the majority of studies (38/43, 88.4%) used ordinary acupuncture (body and scalp acupuncture) (31–40, 42–47, 49–54, 56–71), while three studies used warm acupuncture (30, 41, 48), and the other two studies used electroacupuncture (29, 55).

#### 3.2.3 The usage frequency of acupuncture points

**Figure 2:**
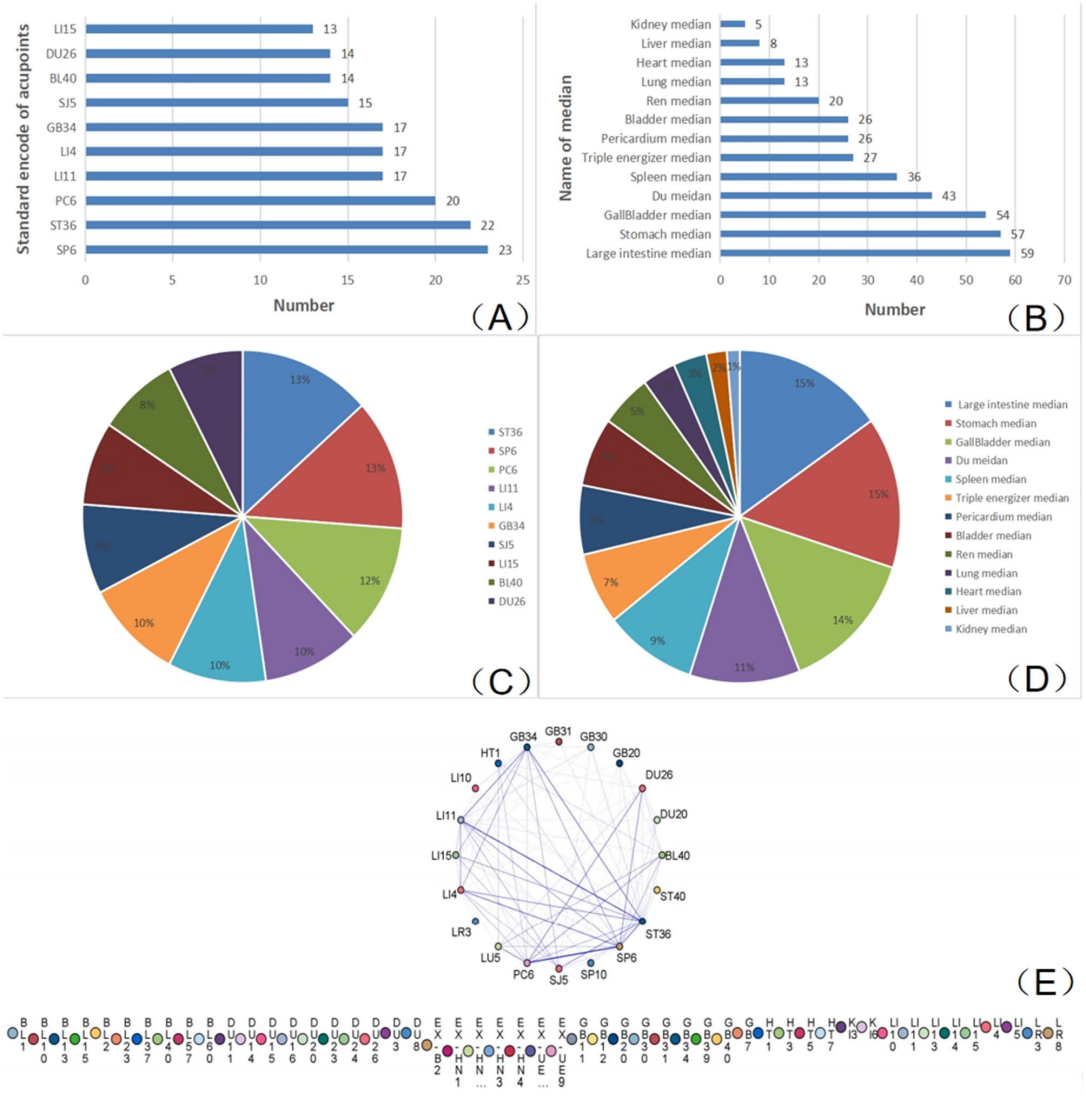
Frequency of usage for acupuncture points and meridians. **(A)** The 10 most frequently used acupuncture points; **(B)** the frequency of each meridian; **(C)** the proportion of the 10 most frequently used acupuncture points; **(D)** the proportion of the frequency for each meridian; and **(E)** Network diagram illustrating the combination rules for selected acupuncture points.

A total of 101 acupoints were included for the treatment of CI, with a total frequency of 402 applications. All 101 acupuncture points belonged to 11 regular meridians, including the Ren meridian, Du meridian, and extra-acupuncture points. Among all acupoints, the top 10 most frequently used were SP6, ST36, PC6, LI11, LI4, GB34, SJ5, BL40, DU26, and LI15, accounting for 5.72%, 5.47%, 4.98%, 4.23%, 4.23%, 4.23%, 3.73%, 3.48%, 3.48%, and 3.23%, respectively (Figure 2C). These points mainly belong to the meridians of the large intestine, the stomach, and the gallbladder, accounting for 15%, 15%, and 14%, respectively (Table 2, Figures 2B, D).

**Table 2:** Combinations rules of acupuncture points across multi-meridians.

**Double association**			**Triple association**		
**Combined acupuncture points**	**Support threshold**	**Confidence threshold**	**Combined acupuncture points**	**Support threshold**	**Confidence threshold**
PC6 → LU5	30.23	100	PC6 → LU5+BL40	25.58	100
LU5 → HT1	23.26	100	PC6 → LU5+SP6	25.58	100
PC6 → HT1	23.26	100	PC6 → BL40+SP6	25.58	100
ST36 → GB30	20.93	100	PC6 → HT1+LU5	23.26	100
GB34 → LI10	18.60	100	LU5 → HT1+PC6	23.26	100
GB34 → GB31	16.28	100	SP6 → LI4+PC6	23.26	100
ST36 → GB31	16.28	100	LU5 → HT1+BL40	20.93	100
LI4 → LR3	13.95	100	LU5 → HT1+SP6	20.93	100
ST36 → LI11	39.53	94.12	PC6 → HT1+BL40	20.93	100
PC6 → DU26	32.56	92.86	PC6 → HT1+SP6	20.93	100

The high frequency of acupoints such as SP6, ST36, and PC6 plays an important role in the treatment of CI. SP6, located on the Spleen Meridian, is commonly used to regulate blood circulation and nourish Yin, which is essential for stroke recovery (72). ST36, a classic point on the Stomach Meridian, plays a vital role in strengthening the body's constitution and improving limb function (73). PC6, along the Pericardium Meridian, is closely related to cardiovascular and cerebrovascular diseases and is frequently used to regulate Qi, calm the mind, and enhance cerebral blood flow (74). These points, along with others in the large intestine meridian, gallbladder meridian, and stomach meridian, collectively demonstrate that acupoint selection for cerebral infarction often emphasizes promoting circulation, dispelling blood stasis, and supporting the recovery of motor and sensory function.

These points, along with others in the large intestine, gallbladder, and stomach Meridians, collectively demonstrate that acupoint selection for CI often emphasizes promoting circulation, dispelling stasis, and supporting the recovery of motor and sensory functions.

#### 3.2.4 The combinations rule for selecting acupuncture points in CI treatment

By performing the Apriori algorithm from a holistic view, we discovered that PC6 (pericardium meridian), LU5 (lung meridian), HT1 (heart meridian), DU26 (Du meridian), SP6 (spleen meridian), BL40 (bladder meridian), ST36 (stomach meridian), LI11 (large intestine meridian), GB34 (gallbladder meridian), and LI10 (large intestine meridian) were frequently combined for CI treatment (Figure 2E). Additionally, the double combinative results (Table 2) showed that PC6 with LU5, LU5 with HT1, and PC6 with HT1 were usually double combined in practice. Furthermore, the combinations of PC6 with LU5+BL40, PC6 with LU5+SP6, PC6 with BL40+SP6, PC6 with HT1+LU5, LU5 with HT1+PC6, SP6 with LI4+PC6, LU5 with HT1+BL40, LU5 with HT1+SP6, PC6 with HT1+BL40, and PC6 with HT1+SP6 were usually triple combined for CI treatment.

### 3.3 Risk of bias assessment

**Figure 3:**
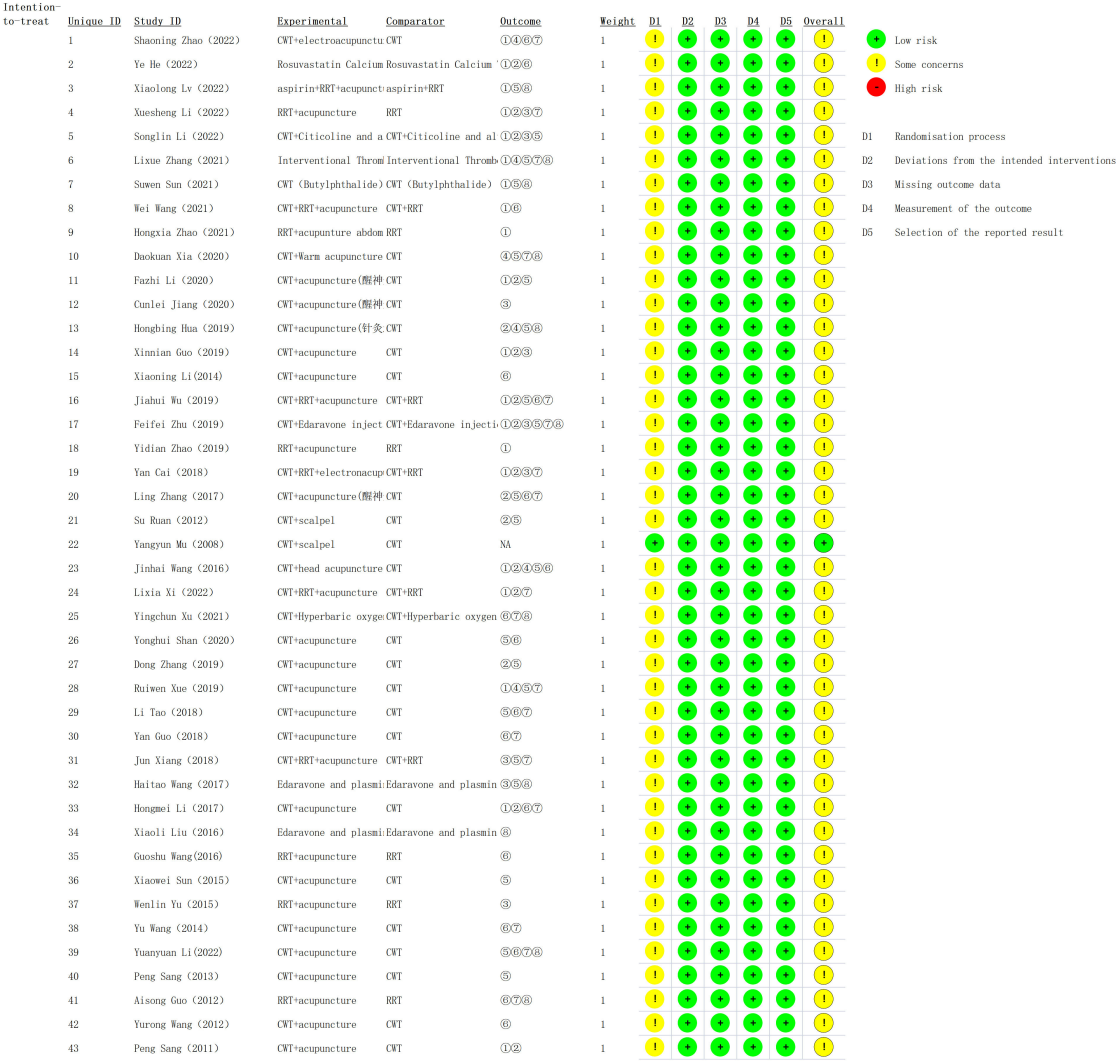
Results of the risk-of-bias assessment using ROB2.

The majority of studies (42/out of 43, 97.7%) did not report details of allocation concealment or implement blinding (29–70), which was categorized as having “some concerns” in D1. However, only one study was single-blind and was considered a “low risk” in D1 (71). All studies published complete data, and the risk of missing outcome data was rated as “low” in D2. According to the expected outcome, no bias was found in any of the RCTs, and the risk of deviation was classified as “low” in D3. Although the majority of studies (42 out of 43, 97.7%) did not mention blinding, the assessment of outcomes was not affected by knowledge of the intervention received. Therefore, the risks of outcome measurement and selection bias in reporting outcomes were considered “low” in D4 and D5, as they were not observed in any of the RCTs. Figure 3 illustrates the results of the risk of bias for the included RCTs.

### 3.4 Meta-analysis results

#### 3.4.1 Primary outcome of TNF-α

**Figure 4:**
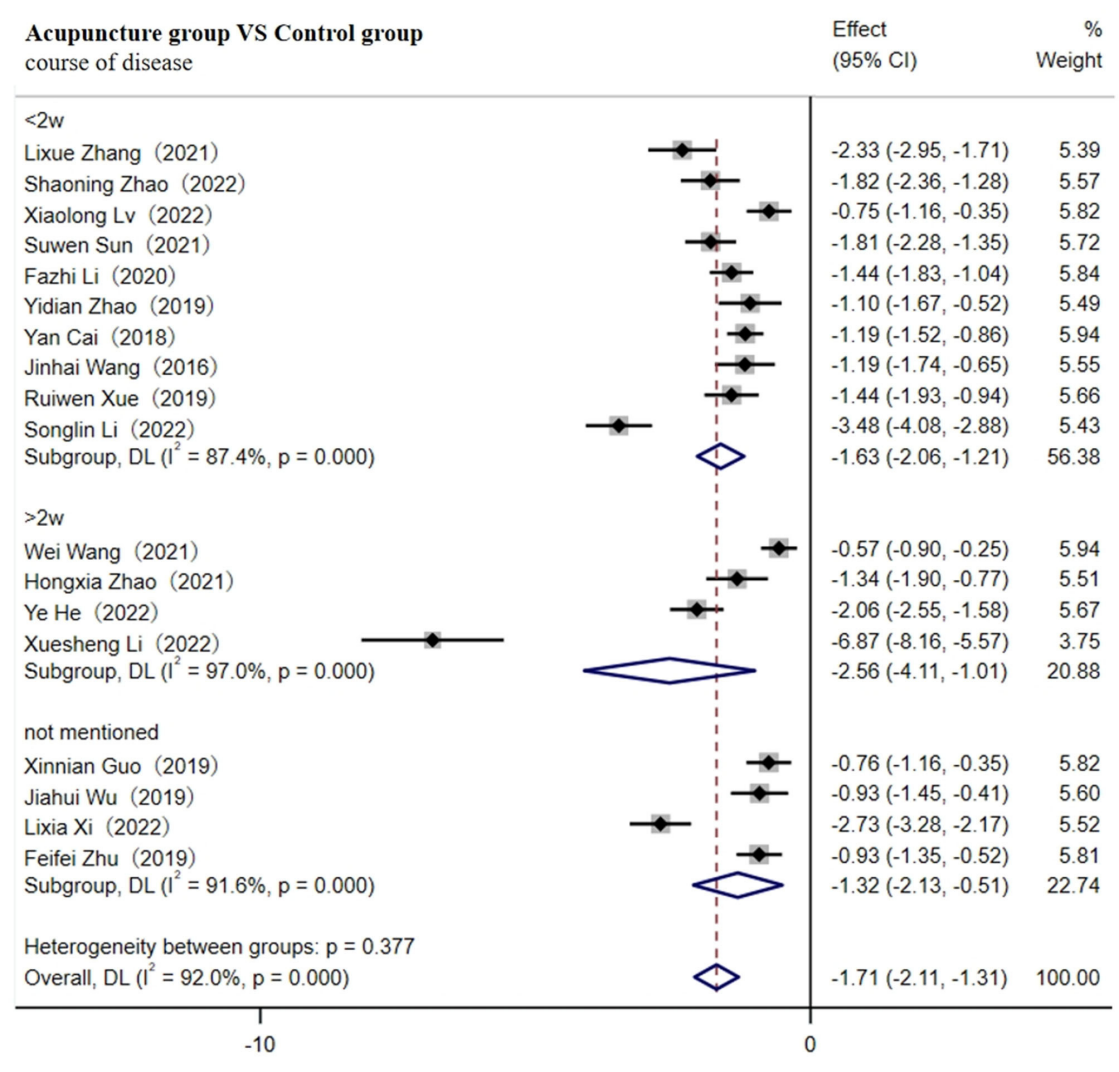
Forest plot of TNF-α (subgroup based on the course of the disease).

A total of 18 studies involving 1,673 patients reported levels of TNF-α (29– 31, 33–37, 39, 40, 43, 46–48, 50, 51, 55, 59). A random-effects model was used for meta-analysis due to the high heterogeneity between studies (I^2^ ≥ 75%). The results showed that the course of disease lasting <2w [SMD = −1.63; 95% CI(−2.06, −1.21); *p* < 0.001], more than 2w [SMD = −2.56; 95% CI (−4.11, −1.01); *p* < 0.001), or not mentioned (SMD = −1.32; 95% CI (−2.13, −0.51); *p* < 0.001] all resulted in lower levels of TNF-α in the acupuncture group (Figure 4). Heterogeneity decreased to 31.6% after removing nine studies through sensitivity analysis [SMD = −1.36; 95% CI (−1.51, −1.20); *p* < 0.001, Supplementary Figure 1].

As shown in Table 1, compared with other studies, the duration of four studies was more than 1 month (31, 33, 34, 39). One study did not report the needle retention time (46). The retained time of acupuncture treatment in two studies was the shortest (20 min) (35, 36), and the method of acupuncture in two studies was warm acupuncture (30, 48), which might contribute to the high heterogeneity observed. Meta-regression analysis verified that duration and needle retention time may be the main sources of heterogeneity (*p* < 0.05, as shown in Table 3). Therefore, subgroup analysis according to duration (≤1 month, 1–2 months, or ≥2 months) and retention time (≤20 min, 20–40 min, ≥40 min, or not mentioned) both showed that the adjunctive use of acupuncture significantly reduced the level of TNF-α better compared to the control group.

**Table 3:** Meta-regression analysis of TNF-α results.

**_ES**	**Coefficient**	**Std. err**.	***t***	**P>|t|**	**[95% conf. interval]**	
Method of acupuncture	−0.0365794	0.3838867	−0.10	0.925	−0.8599345	0.7867757
Needle retention time	0.8554381	0.3623035	2.36	0.033	0.0783745	1.632502
Duration	−1.337167	0.4915722	−2.72	0.017	−2.391485	−0.2828496
_cons	−1.819305	1.261501	−1.44	0.171	−4.524955	0.8863445

The method of acupuncture was categorized as 1, 2, and 3 when the outcome measure unit was warm acupuncture, electroacupuncture, or.ug/ml and others; needle retention time was categorized as 1, 2, 3, and 4 when the needle retention times were 20 min, 30 min, ≥40 min, and not mentioned; duration was categorized as 1, 2, and 3 when duration was <1 month, 1–2 months, and ≥ 2 months.

#### 3.4.2 Primary outcome measures of IL-6

**Figure 5:**
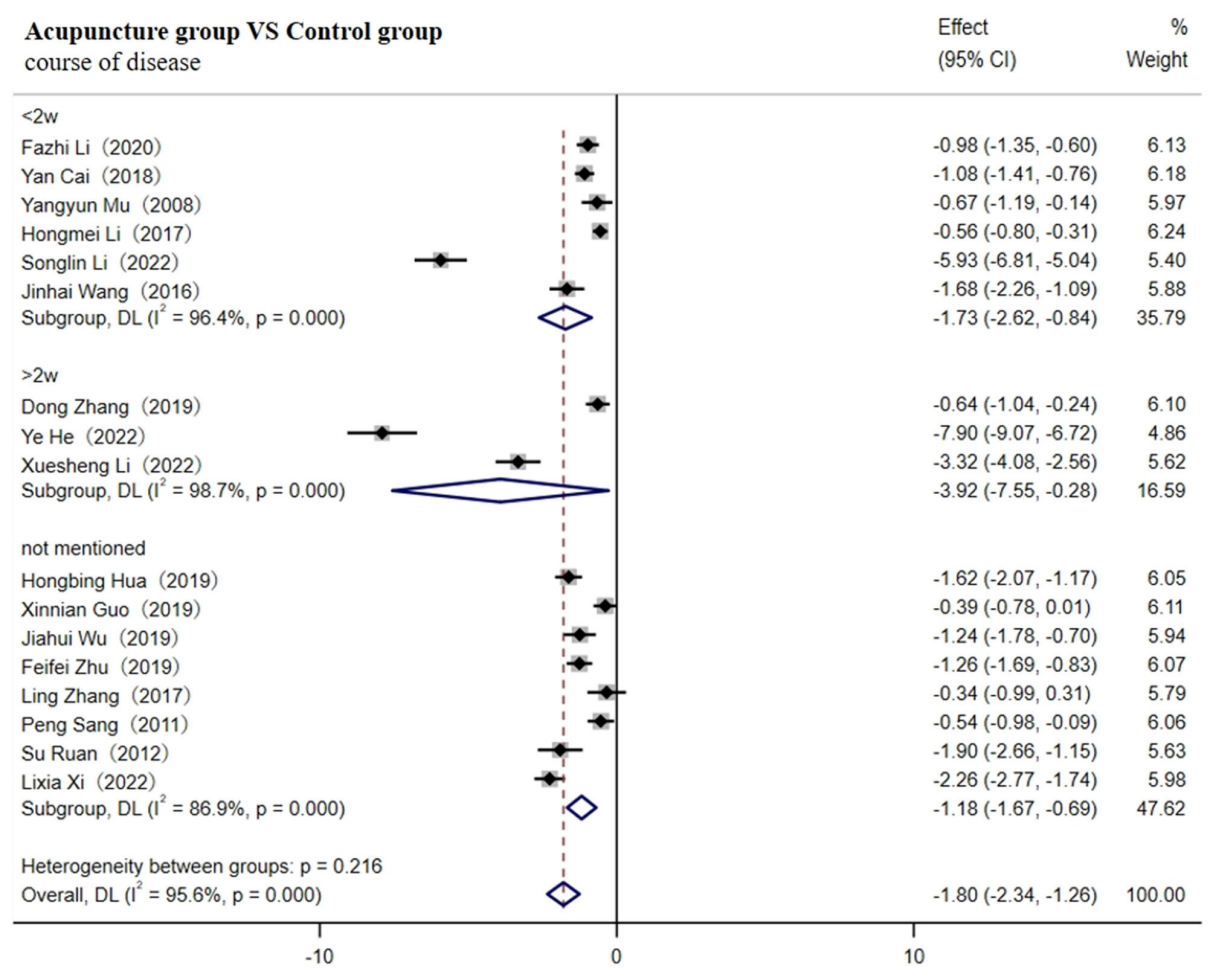
Forest plot of IL-6 (subgroup based on the course of the disease).

A total of 17 studies involving 1,560 patients reported the level of IL-6 (30, 33–35, 43, 45–49, 55, 57–59, 67, 70, 71). The random-effects model was used for the meta-analysis due to high heterogeneity between studies (I^2^ ≥ 75%). As shown in Figure 5, the results indicated that the duration of the disease for <2w [SMD = −1.73; 95% CI (−2.62, −0.84); *p* < 0.001], more than 2w [SMD = −3.92; 95% CI (−7.55, −0.28); *p* < 0.001], or not mentioned [SMD = −1.18; 95% CI (−1.67, −0.69); *p* < 0.001] all resulted in a greater decrease in the level of IL-6 in the acupuncture group (Figure 5). Heterogeneity was reduced to 71.6% after removing six studies through sensitivity analysis [SMD = −0.82; 95% CI (−0.94, −0.70); *p* < 0.001, Supplementary Figure 2].

As shown in Table 1, the duration of two studies was more than 1 month, while one study had the shortest duration (7d) (33, 34, 59). The acupuncture treatment retention time in the two studies was also the shortest (20 min) (35, 67). Additionally, the intervention in one study involved only the use of Rosuvastatin Calcium Tablets (30), which may contribute to the high heterogeneity. Meta-regression analysis indicated that the intervention might be the main source of heterogeneity (*p* < 0.05, as shown in Table 4).

**Table 4:** Meta-regression analysis of IL-6 results.

**_ES**	**Coefficient**	**Std. err**.	***t***	**P>|t|**	**[95% conf. interval]**	
Intervention	−3.851879	1.241915	−3.10	0.008	−6.534873	−1.168886
Duration	−0.0052949	0.5138316	−0.01	0.992	−1.115361	1.104771
Needle retained time	0.7608274	0.4005648	1.90	0.080	−0.1045402	1.626195
_cons	0.7013852	1.850686	0.38	0.711	−3.296778	4.699548

Interventions were categorized as 1, 2, and 3 based on the type of intervention: conventional Western treatment, use of only Rosuvastatin Calcium Tablets, or regular rehabilitation training. The needle retention time was categorized as 1, 2, 3, and 4 when the retention times were 20 min, 30 min, ≥40 min, and not mentioned, respectively. Duration was categorized as 1, 2, 3, and 4, corresponding to durations of ≤7 days, 8–15 days, 16–30 days, and >1 month.

Subgroup analysis based on intervention (CWT, Rosuvastatin Calcium Tablets, or regular rehabilitation training) showed that the adjunctive use of acupuncture significantly decreased the level of IL-6 compared to the control group.

#### 3.4.3 Primary outcome measures of hs-CRP

A total of 17 studies involving 1,636 patients reported hs-CRP levels (29, 30, 32, 38, 39, 42, 47, 52, 53, 57–59, 61, 64, 65, 68, 69). The random-effects model was used for the meta-analysis due to the high heterogeneity among the studies (I^2^ ≥ 75%).

**Figure 6:**
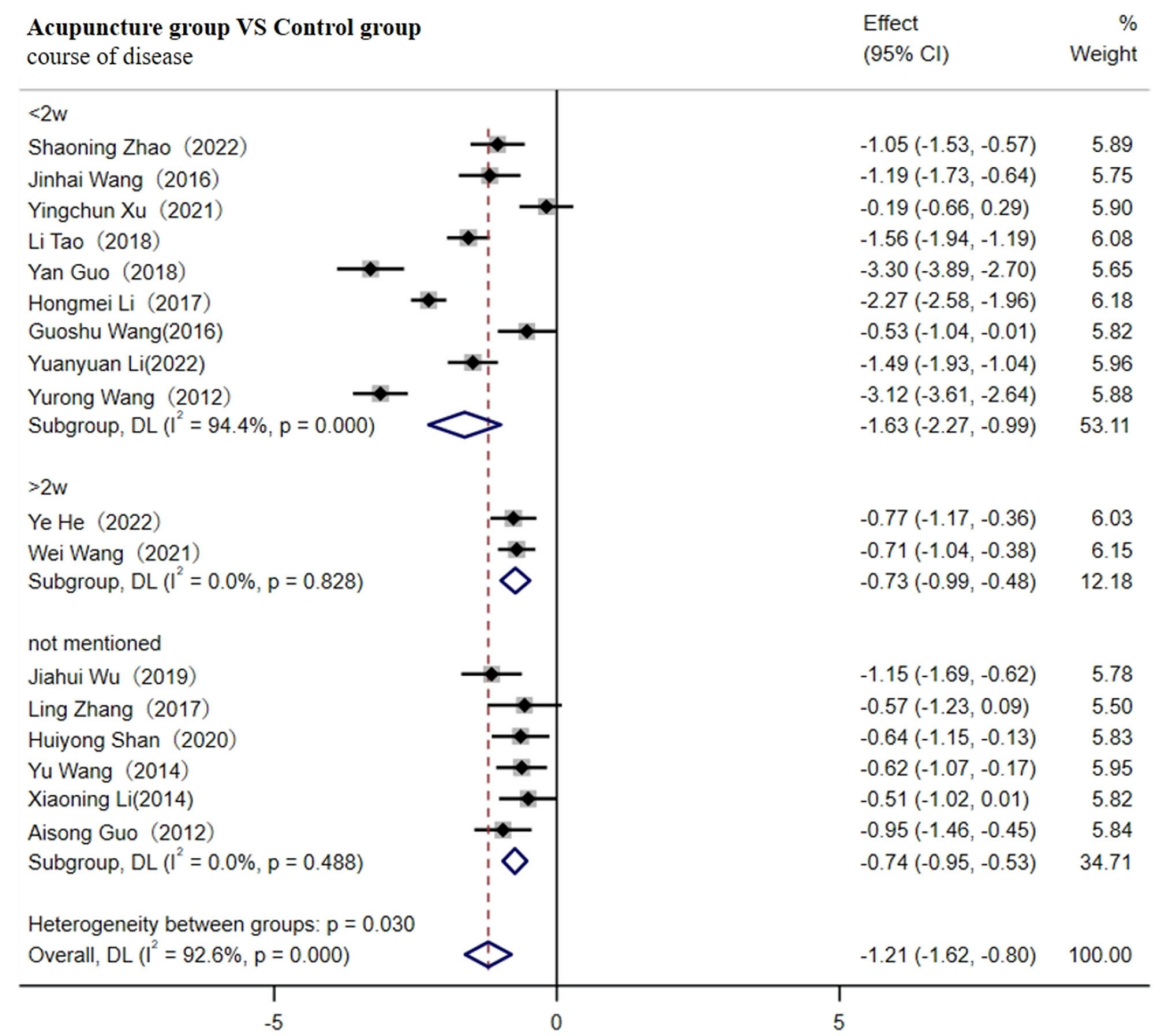
Forest plot of hs-CRP (subgroup based on the course of the disease).

As shown in Figure 6, the results indicate that a disease course of < 2 w [SMD =-1.63; 95% CI (−2.27, −0.99); *p* < 0.001], more than 2 w [SMD = −0.73; 95% CI(−0.99, −0.48); *p* < 0.001], or cases not mentioned [SMD = −0.74; 95% CI(−0.95, −0.53); *p* < 0.001] all resulted in a greater decrease in hs-CRP levels in the acupuncture group (Figure 6).

The heterogeneity decreased to 68.1% after the removal of four studies through sensitivity analysis [SMD = −0.86; 95% CI(−0.99, −0.74); *P* < 0.001, Supplementary Figure 3]. As shown in Table 1, one study had the youngest average age (69), the duration of two studies was the shortest (53, 59), and one study had a sample size exceeding 200 (58), which may contribute to the heterogeneity. Meta-regression analysis indicated that sample size could be the main source of heterogeneity (*p* < 0.05, as shown in Table 5). Consequently, subgroup analysis indicated that sample sizes of <100, between 100 and 149, or more than 150 all indicated that the adjunctive use of acupuncture significantly reduced the level of hs-CRP compared to the control group.

**Table 5:** Meta-regression analysis of hs-CRP results.

**_ES**	**Coefficient**	**Std. err**.	***t***	**P>|t|**	**[95% conf. interval]**	
Age	−0.0372735	0.3217668	−0.12	0.910	−0.7324084	0.6578614
The sample size	−0.770093	0.2701405	−2.85	0.014	−1.353696	−0.18649
Duration	0.4976336	0.254803	1.95	0.073	−0.0528347	1.048102
_cons	−1.103358	0.8321572	−1.33	0.208	−2.901124	0.6944085

Age was categorized into 1, 2, and 3 when the average age was 60<, 60–70, and not mentioned the average. The sample size was categorized into 1, 2, and 3 when needle retention time was 100<, 100–149, and ≥150. Duration was categorized into 1, 2, 3, and 4 when duration was 7 d≤, 8–15 d, 16–30 d, and >1 month.

#### 3.4.4 Secondary outcome measures of TCER

**Figure 7:**
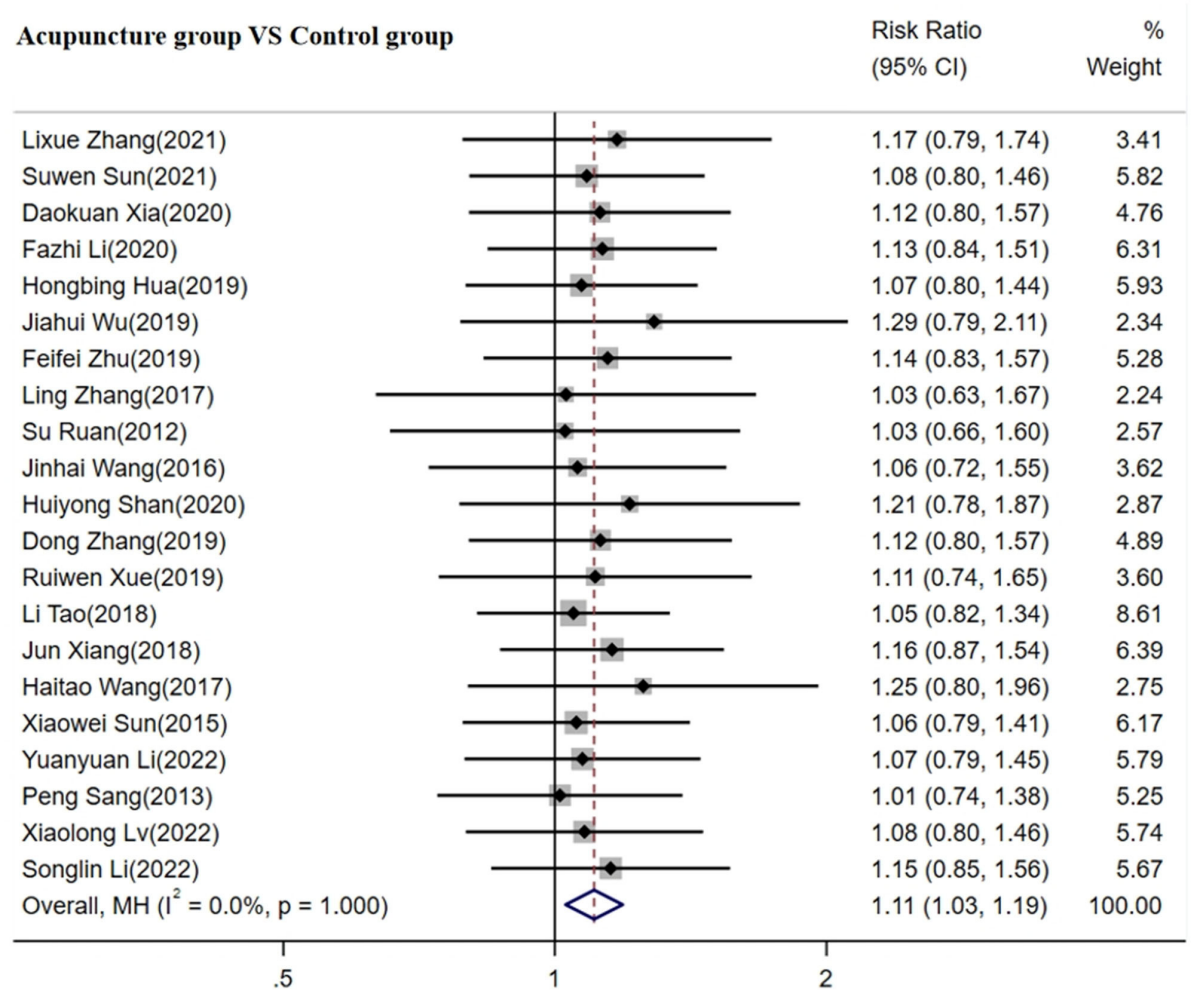
Forest plot of TCER.

A total of 21 studies involving 1,843 patients reported TCE (31, 32, 35–37, 41–43, 45, 47–50, 52, 54, 56, 57, 59, 62, 66, 67). The fixed-effects model was used for the meta-analysis due to minimal heterogeneity between the studies (*p* < 0.05, I^2^ = 0%). As shown in Figure 7, the meta-analysis results indicated that acupuncture was more effective in improving the TCER compared to the control group [RR =1.11; 95% CI(1.03, 1.19); *p* < 0.05].

#### 3.4.5 Secondary outcome measures of NIHSS scores

**Figure 8:**
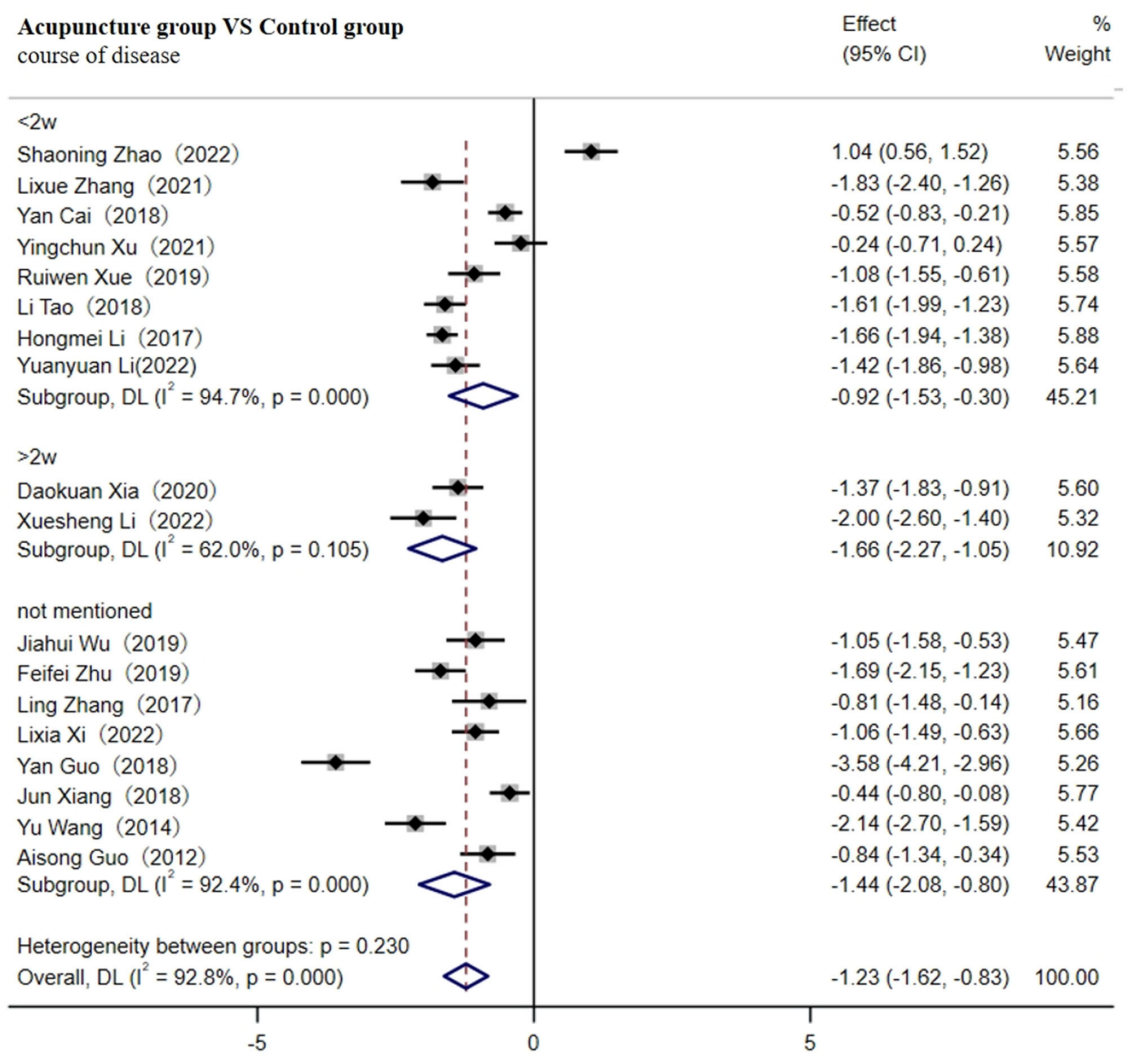
Forest plot of NIHSS scores (subgroup based on the course of the disease).

A total of 18 studies involving 1,782 patients reported the NIHSS scores (29, 32–34, 36, 38, 41, 47, 48, 50, 52–55, 57, 58, 63, 68). A random-effects model was used for meta-analysis due to the high heterogeneity between studies [*p* < 0.001, (I^2^ ≥ 75%)]. The results of the meta-analysis indicated that acupuncture significantly decreased the NIHSS scores [SMD = −1.23; 95% CI (−1.62, −0.83); *p* < 0.001, Supplementary Figure 4]. Sensitivity analysis found no sources of high heterogeneity. As shown in Figure 8, subgroup analysis revealed that whether the course of CI was within 2 w [SMD = −0.92; 95% CI (−1.53, −0.30); *p* < 0.001], more than 2 w (SMD = −1.66; 95% CI (−2.27, −1.05); *p* < 0.001], or not mentioned [SMD = −1.44; 95% CI (−2.08, −0.80); *p* < 0.001], acupuncture significantly decreased NIHSS scores compared to the control group. However, high-quality future RCTs with large samples are needed to confirm this meta-analysis result, as we could not rule out high heterogeneity through sensitivity analysis, meta-regression, or subgroup analysis.

#### 3.4.6 Secondary outcome measures of CRP

Nine studies involving 823 patients reported the level of CRP (33, 35, 44, 46, 48, 54–56, 63). The random-effects model was used for meta-analysis due to high heterogeneity among the studies (I^2^ ≥ 75%). The meta-analysis results indicated that acupuncture significantly reduced CRP levels more effectively [SMD = −3.16; 95% CI (−4.08,−2.24); *p* < 0.001, Supplementary Figure 5].

**Figure 9:**
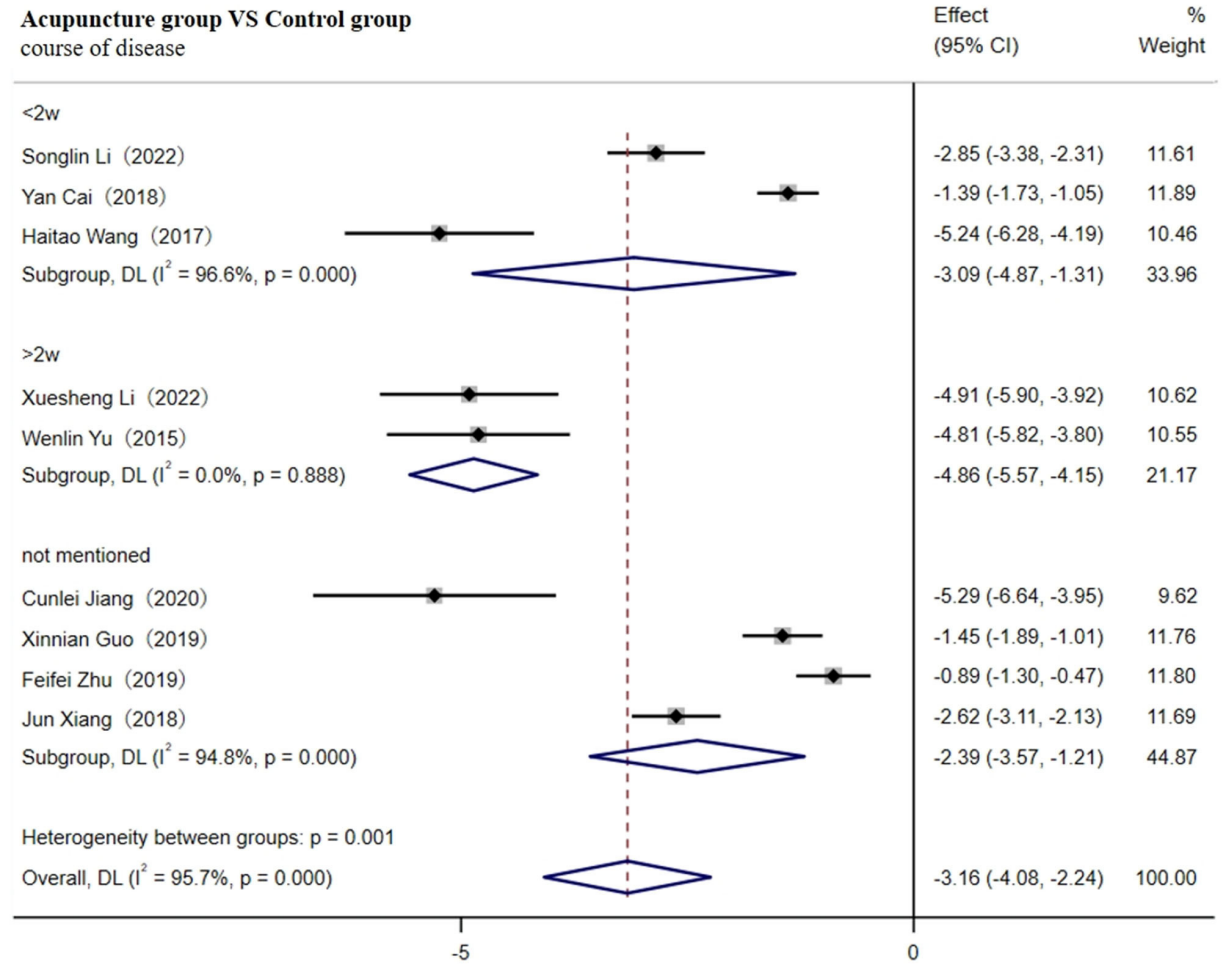
Forest plot of CRP (subgroup based on the course of the disease).

Sensitivity analysis found no sources of high heterogeneity. As shown in Figure 9, subgroup analysis according to the course of the disease indicated that regardless of whether the duration was within 2 w [SMD = −3.09; 95% CI(-4.87, −1.31); *p* < 0.001], more than 2w [SMD = −4.86; 95% CI(−5.57, −4.15); *p* < 0.001], or not mentioned [SMD = −2.39; 95% CI(−3.57, −1.21); *p* < 0.001], acupuncture reduced the level of CRP more effectively compared to the control group. However, future high-quality RCTs with large samples are necessary to confirm this meta-analysis result, as we could not rule out the high heterogeneity through sensitivity analysis, meta-regression, or subgroup analysis.

#### 3.4.7 Secondary outcome measures of IL−1β

**Figure 10:**
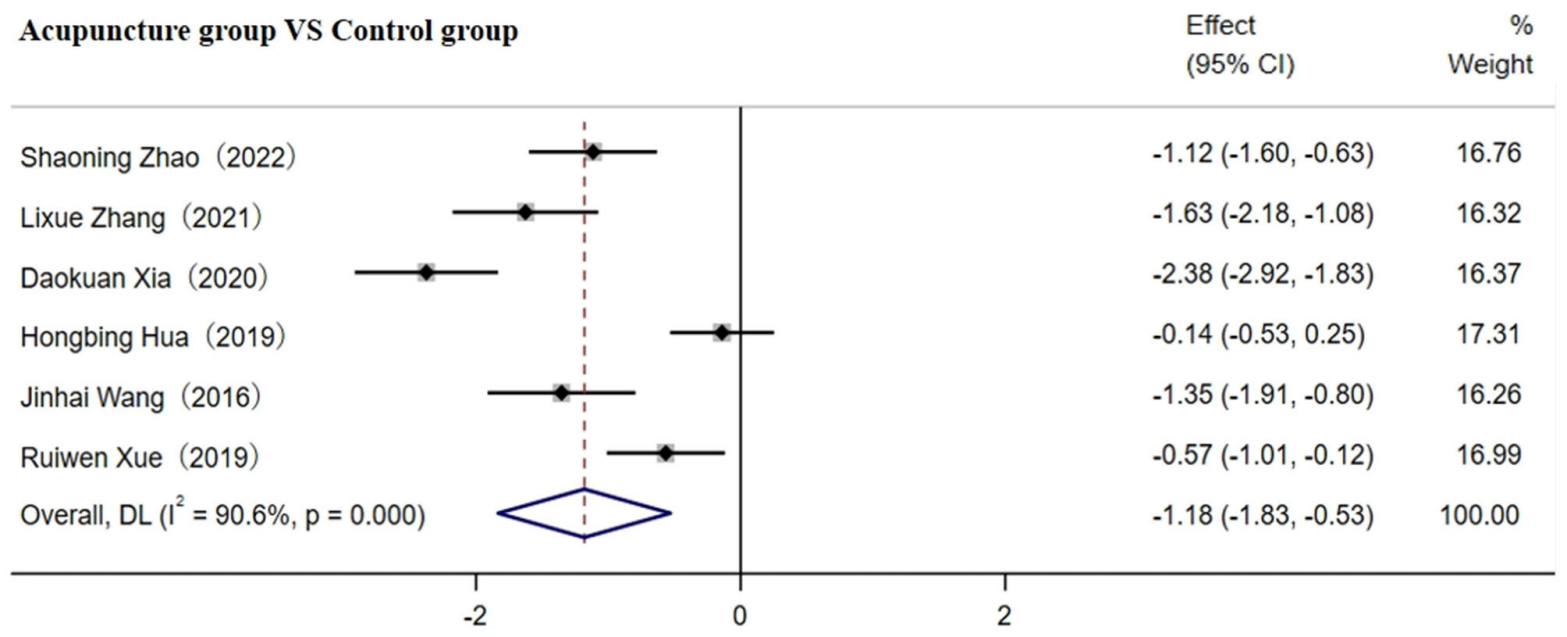
Forest plot of IL-1β before sensitivity analysis.

Six studies involving 475 patients reported on IL-1β (29, 36, 41, 45, 50, 59). The random-effects model was used for the meta-analysis due to high heterogeneity among the studies (I^2^ ≥ 75%). As shown in Figure 10, the meta-analysis results indicated that acupuncture significantly reduced CRP more effectively [SMD = −1.18; 95% CI(−1.83,−0.53); *p* < 0.001].

After removing two studies, heterogeneity between studies was significantly reduced to 69.4%. As shown in Table 1, the disease course in one study was more than 2 w, while another study did not specify this (41, 45), which may contribute to the high heterogeneity observed. The results indicated that IL-1β levels in o CI patients were significantly reduced by the adjunctive use of acupuncture plus CWT [SMD = −1.10; 95% CI (−1.35, −0.85); *p* < 0.001, Supplementary Figure 6]. However, future high-quality RCTs with larger samples are required to confirm this meta-analysis result, as we could not rule out the high heterogeneity through subgroup analysis due to the small number of included studies.

### 3.5 Safety of AE comparison

**Figure 11:**
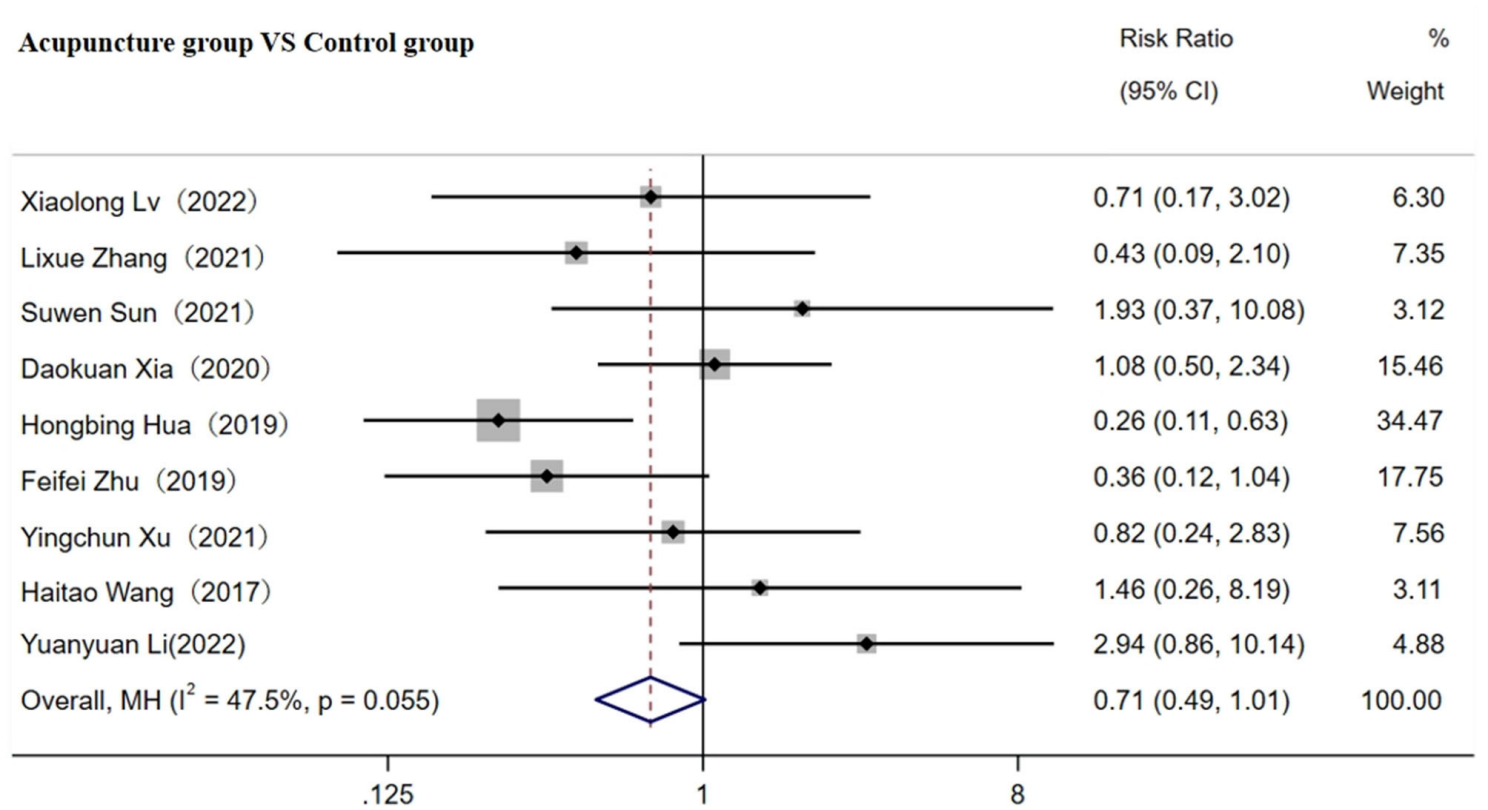
Forest plot comparing various adverse events.

A total of 11 studies involving 925 patients reported the adverse event (AE) rate (31, 32, 36–38, 41, 45, 48, 56, 60, 68). A fixed-effects model was used for the meta-analysis due to low heterogeneity among studies (*p* < 0.05, I^2^ = 47.5%). The results indicated that acupuncture reduced the AE rate compared to the control group [RR = 0.71; 95% CI (0.49, 0.11); *p* < 0.001, see Figure 11].

### 3.6 Results of publication bias assessment

**Figure 12:**
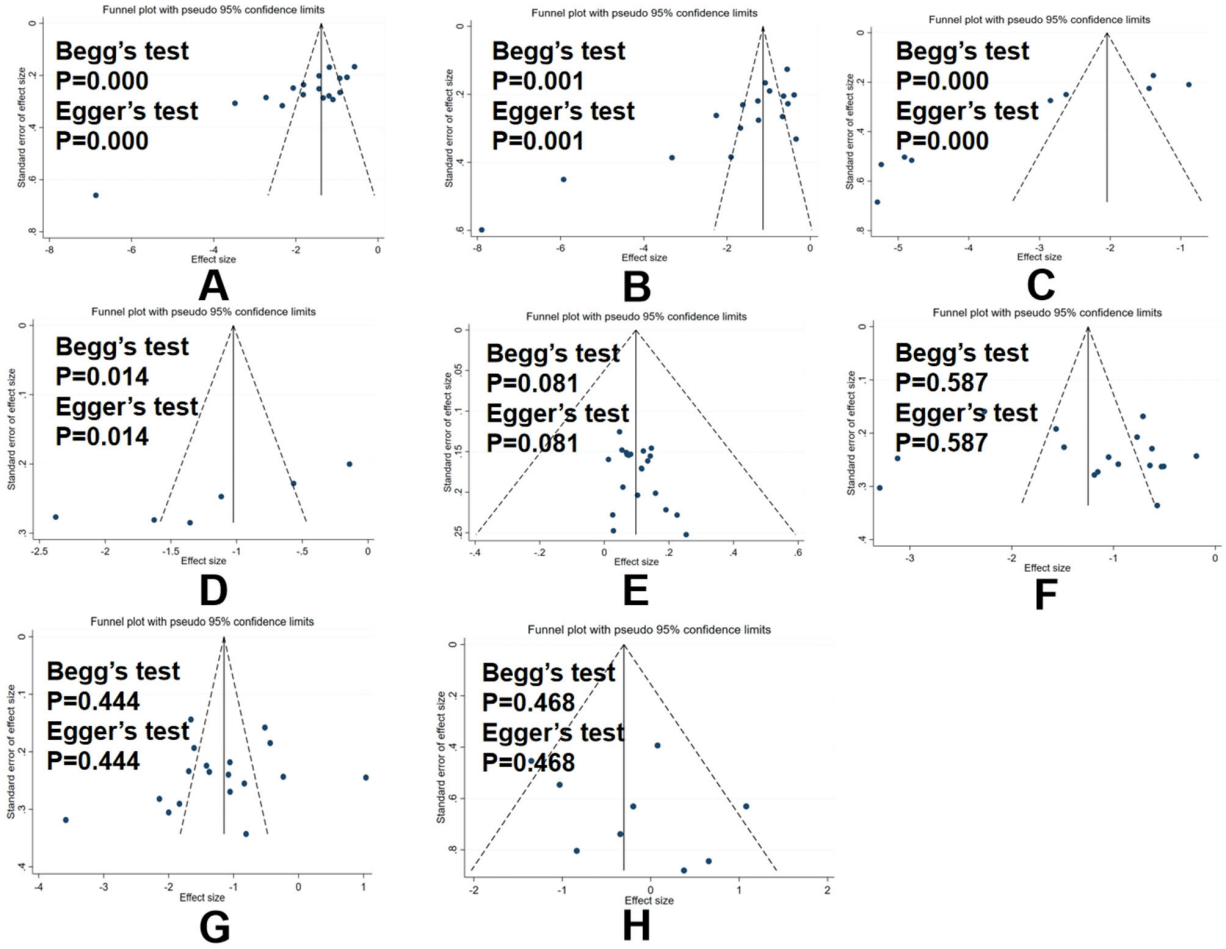
Funnel plot assessing publication bias. **(A)** publication bias assessment for TNF-α; **(B)** publication bias assessment for IL-6; **(C)** publication bias assessment for hs-CRP; **(D)** publication bias assessment for TCER; **(E)** publication bias assessment for NIHSS scores; **(F)** publication bias assessment for CRP; **(G)** publication bias assessment for IL-1β; **(H)** and publication bias assessment for AE rate.

We assessed publication bias regarding the results of TNF-α, CRP, IL−1β, IL-6, TCER, hs-CRP, NIHSS scores, and AE rate. As shown in Figure 12, the Egger and Begg analyses suggested that publication bias existed in the results of TNF-α, IL-6, and hs-CRP (*p* < 0.05), while they indicated that no publication bias existed in the results of TCER, NIHSS scores, CRP, IL-1β, and AE rate (*p* > 0.05). However, due to the lack of access to clinical trial registration or study protocol information, the possibility of selective reporting of results cannot be ruled out (detailed in Figure 12).

### 3.7 Results of trial sequential analysis

**Figure 13:**
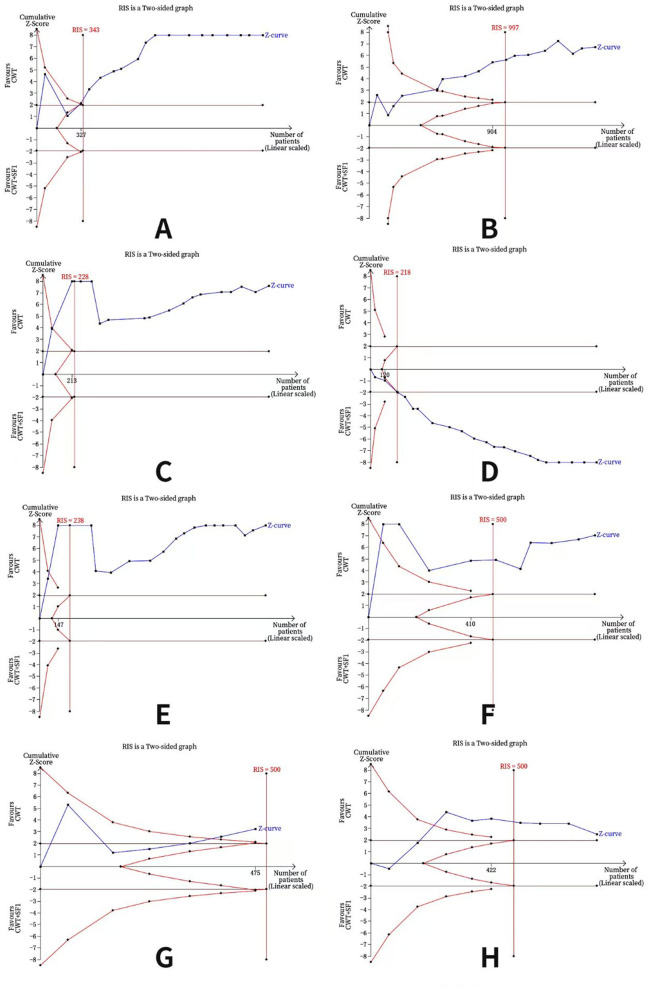
Results of TSA **(A)** TNF-α, **(B)** IL-6, **(C)** hs-CRP, **(D)** TCER, **(E)** NIHSS scores, **(F)** CRP, **(G)** IL-1β, **(H)** AE rate; RIS, required information size.

Regarding TNF-α, CRP, IL-6, TCER, hs-CRP, NIHSS scores, and AE rate, the curve crossed both the traditional and TSA bounds, and the cumulative amount of information reached the expected value, indicating that the current accumulated evidence for acupuncture in the treatment of CI has a sufficient sample size, allowing for a strong conclusion on the results without the need for further clinical trials in the future. Moreover, concerning IL-1β, the curve crossed both the traditional and TSA bounds, but the cumulative amount of information did not reach the expected value. A positive conclusion regarding the adjunctive use of acupuncture in reducing IL-1β compared to CWT alone was therefore reached in advance without additional tests (detailed in Figure 13).

### 3.8 The quality of the evidence

The certainty of evidence regarding meta-outcomes was evaluated using the Grading of Recommendations Assessment, Development, and Evaluation (GRADE) methods, which consider the risk of bias, inconsistency, indirectness, imprecision, and publication bias. The results for TCER and TNF-α were assessed as “high” in terms of certainty of evidence, while IL-6 and hs-CRP were rated as “moderate” due to serious risks of bias, serious inconsistency, and strong associations. The AE rate was also rated as “moderate,” owing to serious bias risks, serious indirectness, and strong associations. Furthermore, the certainty of evidence for CRP was assessed as “moderate” due to serious risks of bias, very serious inconsistency, and very strong associations. The NIHSS scores were rated as “low ” because of serious bias risks, very serious inconsistency, serious indirectness, and very strong associations. Only IL-1β was rated as “very low” in evidentiary quality due to serious risk of bias, serious inconsistency, serious imprecision, and serious indirectness (Table 6).

**Table 6:** Summary of findings based on the grading of recommendations assessment, development, and evaluation (GRADE) method.

**Certainty assessment**							**Summary of findings**			**Comments**
**Participants (studies) Follow-up**	**Risk of bias**	**Inconsistency**	**Indirectness**	**Imprecision**	**Other considerations**	**Overall certainty of evidence**	**Events**		**Anticipated absolute effects or relative effects (95% CI)**	
							**Control**	**Experiment**		
TNF-α 786 (9 RCTs)	Serious^a^	Not serious	Not serious	Not serious	Strong association	⊕⊕⊕⊕ High	392	394	SMD 1.36 lower (1.51 lower to 1.20 lower)	Risk of bias (−1^a^)
IL-6 1190(17 RCTs)	Serious^a^	Serious^c^	Not serious	Not serious	Strong association	⊕⊕⊕○ Moderate	593	597	SMD 0.82 lower (0.94 lower to 0.70 lower)	Risk of bias(−1^a^) Inconsistency(−1^c^)
hs-CRP 1065 (13 RCTs)	Serious^a^	Serious^c^	Not serious	Not serious	Strong association	⊕⊕⊕○ Moderate	532	533	SMD 0.86 lower (0.99 lower to 0.74 lower)	Risk of bias(−1^a^) Inconsistency(−1^c^)
Total effective rate 1843 (21 RCTs)	Serious^a^	Not serious	Not serious	Not serious	Very strong association	⊕⊕⊕⊕ High	716/917 (78.1%)	868/926 (93.7%)	RR 1.11 (1.03 to 1.19)	Risk of bias(−1^a^)
NIHSS scores 1782 (18 RCTs)	Serious^a^	Very serious^d^	Serious^b^	Not serious	Very strong association	⊕⊕○○ Low	888	894	SMD 1.23 lower (1.62 lower to 0.83 lower)	Risk of bias(−1^a^) Inconsistency(−2^d^) Indirectness(−1^b^)
CRP 823 (9 RCTs)	Serious^a^	Very serious^d^	Not serious	Not serious	Very strong association	⊕⊕⊕○ Moderate	409	414	SMD 3.16 lower (4.08 lower to 2.24 lower)	Risk of bias(−1^a^) Inconsistency(−2^d^)
IL−1β 285 (4 RCTs)	Serious^a^	Serious^c^	Serious^f^	Serious^e^	None	⊕○○○ Very low	142	143	SMD 1.10 lower (1.35 lower to 0.85 lower)	Risk of bias(−1^a^) Inconsistency(−1^c^) Indirectness(−1^e^)
AE rate 1074 (11 RCTs)	Serious^a^	Not serious	Serious^g^	Not serious	Strong association	⊕⊕⊕○ Moderate	70/458 (15.3%)	46/467 (9.9%)	RR 0.71 (0.49 to 1.01)	Risk of bias(−1^a^) Indirectness(−1^g^)

RR, risk ratio; SMD, standardized mean difference. ^a^is an unclear risk of bias, as shown in Table 2; ^b^total of 17 studies showed that the scores in the acupuncture were lower than those in the conventional group, while one study showed the opposite; ^c^the direction of the effect varies, with 50% < I^2^ < 75; ^d^the direction of the effect varies significantly, with 75% < I^2^; ^e^the number of studies was small; ^f^the sample size was too small; ^g^seven studies showed that adverse events in the acupuncture group compared to the conventional group, while four studies reported more.

## 4 Discussion

Acupuncture is a non-drug treatment option for various diseases and plays a significant role in the treatment of CI due to its effective clinical efficacy (75–77). Numerous acupuncture and clinical studies have shown that acupuncture can significantly reduce adverse effects and improve symptoms of CI by reducing the expression of inflammatory factors (78–80).

This systematic review and meta-analysis included 43 RCTs with 3,861 participants, showing that intermittent treatment (5–7 times per week) of acupuncture lasting 2–4 weeks, along with multiple acupoints, may benefit CI treatment. In addition, it suggests that acupuncture may safely exert an anti-inflammatory effect by reducing levels of TNF-α, hs-CRP, and IL-6, which may result in a higher total effective rate and a lower NIHSS score. However, the results should be interpreted with caution for clinical practice until high-quality RCTs are conducted for further confirmation due to the high heterogeneity present in some results and the low quality of some included studies.

### 4.1 The use of acupuncture for CI treatment

The therapeutic effect of acupuncture relates to many factors, including frequency, duration, retention time, acupoint selection, and their combinations, among others (81). This systematic review found that adjunctive acupuncture, with a frequency of 5 to 7 times a week (35/43, 81.4%), a duration of 2–4 weeks (33/43, 76.7%), and a retention time of 30 min (25/43, 58.2%), was primarily chosen for CI treatment. This suggests that intermittent treatment (5–7 times/week, 30 min each session) of adjunctive acupuncture lasting more than 2 w may benefit CI treatment. Acupuncture points are considered pathological and physiologically dynamic entities; the sensitivity of these points changes according to the body's condition and may even diminish in certain situations. This sensitivity is understood as a dynamic process that can be triggered by altered homeostasis and may revert to baseline after acupuncture treatment. In other words, the effectiveness of acupuncture can diminish due to adjustments within the human body, which may explain the non-continuous effects of acupuncture. Therefore, acupuncture treatment with a retention time of 30 min and a treatment duration of at least 14 days (5–7 times/week) may lead to a potential continuous therapeutic effect, ultimately resulting in satisfactory therapeutic outcomes.

However, while these treatment parameters have been validated in RCTs, their application in real-world settings is more complex. In clinical practice, acupuncture treatments may involve patients with varying severities of CI and multiple comorbidities (e.g., hypertension and diabetes), necessitating individualized treatment plans (82, 83). Therefore, although RCTs provide strong evidence for the specific efficacy of acupuncture for CI, their results may not fully reflect the diverse and dynamic nature of real-world clinical practice.

### 4.2 Selection rules for acupuncture points in CI treatment

In addition, ST36, SP6, PC6, LI11, LI4, GB34, SJ5, LI15, DU26, and BL40 were the most frequently chosen acupuncture points for CI treatment. ST36 is a significant tonic acupuncture point according to TCM theory, serving functions such as invigorating the spleen and replenishing qi, strengthening tendons and bones, activating collaterals, and promoting qi in the treatment of CI. ST36 is also confirmed to have anti-inflammatory properties (84).

SP6 strengthens tendons, disperses liver qi, and nourishes the liver and kidneys. PC6, located in the middle of the pericardium, has a calming effect and can regulate qi flow, relieve pain, and stabilize the mind. Combining PC6 and SP6 activates qi and blood flow, dredges the collaterals, reduces swelling, and relieves pain for CI (85). LI11 and LI4 are the most commonly used acupuncture points for movement disorders in CI, as they restore the static motor network and reduce neurological deficits (86).

GB34 can improve the potential of cerebral vasodilation to compensate for fluctuations caused by changes in external conditions, which could potentially be useful for the treatment of CI (87). SJ5 could play a neuroprotective role in CI by regulating the expression of the anti-apoptotic gene Bcl-2 (88). LI15 has been shown to effectively treat symptoms and signs of stroke, especially by improving upper limb motor function and various hemorheological parameters (89, 90). DU26 significantly promotes neuronal function recovery and reduces the expression of inflammatory cytokines, including IL-1β, IL-6, and TNF-α. These effects may be due to its antioxidant, anti-inflammatory, and anti-apoptotic properties (91, 92).

BL40 can reduce the level of inflammation, oxidative stress level, and anti-apoptotic effects in patients with CI (93, 94). The combination of SP6, PC6, and BL40 can play a synergistic effect to enhance their beneficial effects on CI (95). Therefore, in this study, the frequently used acupoints SP6, ST36, PC6, LI11, LI4, GB34, BL40, and DU26 were usually combined due to their synergistic efficacy in the treatment of CI, indicating that the selection and combination of these points (ST36, SP6, PC6, LI11, LI4, GB34, SJ5, LI15, DU26, and BL40) may be beneficial for the treatment of CI through their anti-inflammatory effects.

### 4.3 The anti-inflammatory effects of acupuncture in treating CI

The latest study suggests that acupuncture has satisfactory therapeutic efficacy and safety in the treatment of CI. CI leads to the sudden interruption of blood flow in local or extensive brain tissue, activating the expression of inflammatory cells such as TNF-α, IL-1β, CRP, and IL-6, and causing neutrophils, monocytes, and T cells to accumulate in brain-damaged tissue due to disrupted blood flow to the affected area of the brain (96, 97). Acupuncture has been proven to reduce the production of inflammatory factors, thereby preventing further damage to brain cells caused by inflammation in the treatment of CI (98).

Recent research indicates that acupuncture can regulate the Bcl-2/Bax ratio, which enhances the anti-apoptotic capacity of neural cells, inhibits inflammatory responses, and reduces brain tissue damage in patients with cerebral infarction (99, 100).

Moreover, acupuncture is believed to reduce the inflammatory response and cell damage by increasing cerebral blood flow, promoting the supply of oxygen and nutrients to the infarcted area, and improving the metabolic environment of brain tissue (101, 102).

Additionally, some studies have explored how acupuncture enhances antioxidant capacity by regulating enzymes such as superoxide dismutase (SOD) and glutathione peroxidase (GSH-Px). This process reduces oxidative stress and free radical-induced brain cell damage, inhibits pro-inflammatory pathways, decreases the release of inflammatory mediators, and alleviates neuroinflammation (103, 104).

In this study, we also found that adjunctive acupuncture treatment could decrease the expression levels of TNF-α, IL-1β, CRP, and IL-6, increase the total effective rate, and reduce hs-CRP and NIHSS scores more effectively than the control group. However, the results should be interpreted with caution, as we could not identify the source of the high heterogeneity present in some of the accumulated results, which may be due to differences in literature quality, selected acupoints, acupuncture methods, needle retention time, disease course, age and duration, sample size, or diagnostic criteria among the included RCTs. Meanwhile, emerging perspectives suggest that in real-world trials, although confounding factors contributing to low quality and high heterogeneity are inevitable, their results should still be recommended, as they focus on practical applicability and extrapolation, thereby increasing external validity. Therefore, the findings of this meta-analysis still need to be emphasized (105).

### 4.4 The safety of acupuncture in treating CI

Regarding clinical safety, 15.3% (70/458) of adverse reactions occurred in the control group, compared to 9.9% (46/467) in the acupuncture group. The reported adverse events in the acupuncture group primarily included mild dizziness, headaches, transient rashes, and gastrointestinal discomfort. In contrast, the adverse events in the control group, which mainly involved conventional pharmacological treatments, included more severe complications such as gastrointestinal bleeding, muscle pain, and vascular-related events. Although the *p*-value indicated a significant reduction in AEs with acupuncture, the upper limit of the confidence interval included 1(RR = 0.71; 95% CI 0.49 to 1.01; *p* < 0.05), suggesting that the observed difference was of borderline significance.

CWT for CI mainly targets primary symptoms but may induce additional side effects, especially with long-term use. For instance, vasodilators and antiplatelet agents, commonly used in CI management, have been associated with headaches, muscle pain, gastrointestinal bleeding, and other systemic complications (106–108). Previous studies have also suggested that acupuncture may reduce CI-related complications such as bleeding events, myocardial infarction, functional disability, vascular mortality, vascular events, and all-cause mortality (109–112). It achieves these effects by stimulating specific acupoints, thereby modulating the immune, nervous, and physiological systems. This regulatory mechanism may contribute to a lower incidence of complications (113).

Furthermore, the literature indicates that acupuncture may alleviate gastrointestinal bleeding and related adverse reactions by minimizing stress-induced damage to the gastrointestinal barrier. Additionally, acupuncture's capacity to activate endogenous opioids contributes to its pain-relieving effects, which aligns with our findings (83, 114). Although adjunctive acupuncture may alleviate CI symptoms and reduce adverse events, more high-quality trials are needed to confirm its safety profile.

### 4.5 The assessment of bias risk and confidence in evidence on the meta-results

To validate the credibility of meta-results, we assessed the risk of bias in the included studies. The results indicated that most studies were rated as “some concern” due to a lack of detailed information about the blinding method during randomization, potentially leading to an exaggeration of the effects of reporting bias. Thus, we conducted TSA analysis and GRADE assessment to evaluate our confidence in the results. The TSA analysis showed that a firm, reliable conclusion could be drawn: acupuncture combined with CWT reduced the expression levels of TNF-α, hs-CRP, and IL-6, increased the TCER, and reduced NIHSS scores and AE more effectively than CWT alone, without the need for further tests in the future, because they crossed both the TSA bounds and the cumulative information bound. Additionally, the GRADE assessment indicated that the confidence in the evidence was rated high for TCER, moderate for the evidence of TNF-α, IL-6, and AE, and ranged from very low to low for hs-CRP and NIHSS scores (Table 6). This was mainly due to inconsistent outcomes, varying directions of effect (*I*^2^ > 75%), and serious risks of bias in the included studies.

To further explore potential sources of heterogeneity, we conducted meta-regression and sensitivity analyses. The meta-regression analysis indicated that differences in acupuncture procedures and inconsistent treatment durations might be key sources of heterogeneity (*p* < 0.05). Despite subgroup analysis based on the course of the disease, heterogeneity remained high (I^2^ > 75%), indicating that there was still a large variability between different studies. Possible reasons include differences in acupuncture procedures (such as needling depth, acupoint selection, and use of electroacupuncture vs. manual acupuncture), inconsistent treatment frequency, individual physiological differences, and variations in study methodology. Thus, more RCTs with higher methodological quality are still required to strengthen and update the results further in the future. However, recently, non-blinded pragmatic trials have been recommended for achieving clinically relevant results as they emphasize practical applicability and extrapolation in real-world situations (increased external validity) rather than therapeutic effect, so the reporting of this meta-result still needs to be emphasized (115, 116).

### 4.6 Advantages and limitations of the present study

There were several strengths and demerits worth mentioning in this study. This study was the first systematic review and meta-analysis to summarize and evaluate the anti-inflammatory efficacy of acupuncture for CI using updated PRISMA methods. We found that acupuncture could increase the total effective rate and decrease TNF-α, CRP, IL-6, and adverse effects, which may present an alternative option for physicians when treating CI patients. This study also marked the first time summarizing the rules for acupuncture point selection and application, providing a uniform, standardized, and specific strategy for physicians in practice when treating CI. Additionally, this study was designed in accordance with the high standards of methodological quality set by the updated AMSTAR 2.0, which improved the accuracy, confidence, and clinical applicability of the results (117).

Although the results were encouraging, this study had several drawbacks. First, the duration and needle retention of acupuncture treatment, as well as the control methods used in the included studies, varied; few studies explicitly stated that they performed RCTs with registry information in accordance with the Consolidated Standards of Reporting Trials (CONSORT). Second, the included studies spanned from 2008 to 2021, and the treatment regimens and medications for CI may differ, which could be a primary source of high heterogeneity among studies. Third, the diagnostic criteria in the literature were inconsistent, which may lead to high heterogeneity and publication bias. Fourth, although 43 RCTs were included, the outcome indicators in each study varied, with some indicators represented by only a few RCTs, resulting in insufficient information to eliminate the source of high heterogeneity through sensitivity analysis, meta-regression analysis, or subgroup analysis, among other methods. Fifth, most of the literature was conducted in China, limiting the generalizability and applicability of the results. Sixth, the methodological quality of the included studies was generally low. Specifically, 97.7% (42/43) of the studies did not report adequate details about randomization or allocation concealment, which may introduce bias and affect the reliability of our findings. Additionally, some unpublished or ongoing studies may still be overlooked, potentially leading to publication bias and reducing the robustness of the results. Moreover, due to the low quality of the included studies and the variable quality of the evidence for the results, interpreting the meta-results in clinical practice should be approached with caution until further rigorous trials are designed to validate, strengthen, and update the current study's results. Finally, the methods of acupuncture for CI were divided into three types: ordinary acupuncture, warm acupuncture, and electroacupuncture.

In addition to these limitations, it is important to acknowledge the differences between RCTs and real-world clinical practice. While RCTs provide strong evidence regarding the efficacy of acupuncture under standardized protocols, real-world settings involve diverse patient populations, individualized treatments, and varying levels of practitioner expertise. Observational studies may offer insights into long-term effectiveness and practical applicability, but they are susceptible to bias due to a lack of standardization. Future research should integrate rigorous RCTs with pragmatic, real-world studies to gain a clearer understanding of acupuncture's role in CI treatment.

### 4.7 Implications of prospective research

In the future, clinical RCTs on the anti-inflammatory effect of acupuncture for CI should be improved as follows: (1) During the design phase, the RCTs should be developed strictly according to the CONSORT statements; the methods, treatment details, and outcome indicators should follow standardized and specific guidance or references. (2) When RCTs begin, the methods should be strictly adhered to, and a sufficient sample size should be recruited as estimated in the design phase; the duration and usage of acupuncture should be uniform to reduce heterogeneity between studies. (3) RCTs should have adequate follow-up time to compare the long-term and short-term effects of acupuncture. (4) In the analysis phase, RCTs should report all results to evaluate the efficacy of acupuncture and adequately report any adverse effects.

### 4.8 Comparison with previous meta-analyses and methodological advances

Our findings align with previous meta-analyses on acupuncture for CI, which demonstrated its efficacy in improving neurological function and clinical outcomes (18–21). However, unlike these studies, which primarily focused on clinical efficiency, our study provides a novel perspective by systematically evaluating the anti-inflammatory mechanisms of acupuncture, including its effects on inflammatory markers and related pathways. Additionally, our study addresses the methodological limitations of earlier meta-analyses by incorporating subgroup analysis, sensitivity analysis, and trial sequential analysis (TSA), thereby enhancing the robustness of the results. These advancements offer a more comprehensive understanding of acupuncture's role in CI treatment and highlight the need for future research to further explore its long-term effects and real-world applicability.

## 5 Conclusion

Preliminary evidence suggests that acupuncture may provide anti-inflammatory benefits in treating chronic inflammation. However, due to high heterogeneity and limitations in study quality, further high-quality, multicenter RCTs are required to confirm these findings in the future.

## Data Availability

The original contributions presented in the study are included in the article/Supplementary material; further inquiries can be directed to the corresponding authors.
