# Supplementary material for: Usage, anti-inflammatory effect and safety of adjunctive acupuncture for cerebral infarction: an Apriori algorithm-based data mining and meta-analysis
Source: Front Neurol. 2025 May 19;16:1546194. doi: 10.3389/fneur.2025.1546194 (PMC12128887; doi:10.3389/fneur.2025.1546194)
Supplement: Supplementary file 1 [file Supplementary_file_1.docx]

**Table of contents**

[Supplementary File S1. Search strategies for databases 2](#_Toc13637)

[Supplementary File S2. The details of meta regression.](#_Toc16468) 6

[Supplementary File S3. Lists the characteristic of included RCTs. 1](#_Toc22408)2

[Supplementary File S4. Results of the quality of evidence.](#_Toc22408) 18

[Supplementary File S5. Supplemental Figure.](#_Toc22408) 20

Supplementary File S6. Figure. 25

# Supplementary File S1. Search strategies for databases.

2.1 PubMed (https://pubmed.ncbi.nlm.nih.gov/advanced/)

1. Pubmed database

| Iterm | MeSH | Study number |
| --- | --- | --- |
| #1 | (Acupuncture[Title/Abstract]) | 26088 |
| #2 | (((((Ischemic Encephalopathy[Title/Abstract])) OR (Brain Ischemia[Title/Abstract])) OR (Ischemia stroke[Title/Abstract])) OR (Cerebral infarction[Title/Abstract])) OR (（Cerebral）AND（Ischemic）[Title/Abstract]) | 177218 |
| #3 | ((((((Inflammatory[Title/Abstract])) OR (inflammation[Title/Abstract])) OR (TNF-α[Title/Abstract])) OR (IL-6[Title/Abstract])) OR (IL-1[Title/Abstract])) OR (CRP[Title/Abstract]) | 106086 |
| #4 | #1AND#2AND#3AND(Randomized Controlled Trial[ptyp]) | 6 |

1. Embase database

| Iterm | MeSH | Study number |
| --- | --- | --- |
| #1 | (Acupuncture[Title/Abstract]) | 64130 |
| #2 | (((((Ischemic Encephalopathy[Title/Abstract])) OR (Brain Ischemia[Title/Abstract])) OR (Ischemia stroke[Title/Abstract])) OR (Cerebral infarction[Title/Abstract])) OR (（Cerebral）AND（Ischemic）[Title/Abstract]) | 257977 |
| #3 | ((((((Inflammatory[Title/Abstract])) OR (inflammation[Title/Abstract])) OR (TNF-α[Title/Abstract])) OR (IL-6[Title/Abstract])) OR (IL-1[Title/Abstract])) OR (CRP[Title/Abstract]) | 2076527 |
| #4 | #1AND#2AND#3AND(Randomized Controlled Trial[ptyp]) | 7 |

1. Web of Science

| Iterm | MeSH | Study number |
| --- | --- | --- |
| #1 | TS=(Acupuncture) | 44603 |
| #2 | (((((TS=(Brain Ischemia)) OR TS=(Ischemic Encephalopathy))) OR TS=(Ischemia stroke)) OR TS=(Cerebral infarction)) OR TS=(（Cerebral）AND（Ischemic）) | 247005 |
| #3 | (((((TS=(inflammatory)) OR TS=(inflammation)) OR TS=(TNF-α)) OR TS=(IL-6)) OR TS=(IL-1)) OR TS=(CRP) | 2167846 |
| #4 | #1AND#2AND#3AND(Clinical Trial[ptyp]) | 10 |

Cochrane Library

| Iterm | MeSH | Study number |
| --- | --- | --- |
| #1 | Acupuncture | 19321 |
| #2 | (ischemic AND encephalopathy） OR (brain AND ischemia) OR (ischemia AND stroke) OR (cerebral AND infarction) OR （（cerebral）and（ischemic）） | 17356 |
| #3 | inflammatory OR inflammation OR 'TNF-α' OR 'IL-6' OR 'IL-1' OR CRP | 115092 |
| #4 | #1AND#2AND#3AND(Trial[ptyp]) | 21 |

5、Ovid database

| Iterm | MeSH | Study number |
| --- | --- | --- |
| #1 | (Acupuncture[All Fields]) | 65448 |
| #2 | (((((Ischemic Encephalopathy[All Fields])) OR (Brain Ischemia[All Fields])) OR (Ischemia stroke[All Fields])) OR (Cerebral infarction[All Fields])) OR (（Cerebral）AND（Ischemic）[All Fields]) | 163067 |
| #3 | ((((((Inflammatory[All Fields])) OR (inflammation[All Fields])) OR (TNF-α[All Fields])) OR (IL-6[All Fields])) OR (IL-1[All Fields])) OR (CRP[All Fields]) | 3155962 |
| #4 | （RCT[All Fields]）OR（Random Control Trial[All Fields]）OR（Random Control Trials[All Fields]）OR（Control Trial[All Fields]）OR（Control Trials[All Fields]） | 199767 |
| #5 | #1AND#2AND#3AND#4 | 170 |

6、China National Knowledge Infrastructure (CNKI)) database

| Iterm | MeSH | Study number |
| --- | --- | --- |
| #1 | SU=Acupuncture | 193332 |
| #2 | (((((SU=(Brain Ischemia)) OR SU=(Ischemic Encephalopathy))) OR SU=(Ischemia stroke)) OR SU=(Cerebral infarction)) OR SU=(（Cerebral）AND（Ischemic）) | 210348 |
| #3 | SU=inflammatory OR SU=inflammation OR SU='TNF-α' OR SU='IL-6' OR SU='IL-1' OR SU=CRP | 427259 |
| #4 | #1AND#2AND#3 | 307 |

7、Chinese Biomedical Literature Database (CBM) database

| Iterm | MeSH | Study number |
| --- | --- | --- |
| #1 | Acupuncture[Common fields] | 242127 |
| #2 | (((((Ischemic Encephalopathy[Common fields])) OR (Brain Ischemia[Common fields])) OR (Ischemia stroke[Common fields])) OR (Cerebral infarction[Common fields])) OR (（Cerebral）AND（Ischemic）[Common fields]) | 225009 |
| #3 | ((((((Inflammatory[Common fields])) OR (inflammation[Common fields])) OR (TNF-α[Common fields])) OR (IL-6[Common fields])) OR (IL-1[Common fields])) OR (CRP[Common fields]) | 467061 |
| #4 | #1AND#2AND#3AND(Randomized Controlled Trial[ptyp]) | 202 |

8、Wan-fang Database

| Iterm | MeSH | Study number |
| --- | --- | --- |
| #1 | (Acupuncture[Title/Abstract]) | 201699 |
| #2 | (((((Ischemic Encephalopathy[Title/Abstract])) OR (Brain Ischemia[Title/Abstract])) OR (Ischemia stroke[Title/Abstract])) OR (Cerebral infarction[Title/Abstract])) OR (（Cerebral）AND（Ischemic）[Title/Abstract]) | 224278 |
| #3 | ((((((Inflammatory[Title/Abstract])) OR (inflammation[Title/Abstract])) OR (TNF-α[Title/Abstract])) OR (IL-6[Title/Abstract])) OR (IL-1[Title/Abstract])) OR (CRP[Title/Abstract]) | 373630 |
| #4 | #1AND#2AND#3 | 184 |

9、China Science and Technology Journal Database (VIP)

| Iterm | MeSH | Study number |
| --- | --- | --- |
| #1 | M=Acupuncture | 132926 |
| #2 | M=(ischemic AND encephalopathy） OR (brain AND ischemia) OR (ischemia AND stroke) OR (cerebral AND infarction) OR （（cerebral）and（ischemic）） | 72912 |
| #3 | M=（Inflammatory） OR （inflammation） OR （TNF-α） OR （IL-6） OR (IL-1) OR (CRP) | 155020 |
| #4 | #1AND#2AND#3 | 79 |

# Supplementary File S2. The details of meta regression

**TNF-α**

**Duration** is divided into four levels：

1. 1 month≤；2、1-2 month 3、≥2month

**Needle retention time** is divided into four levels：

1、20 min；2、30 min 3、≥40 min 4、no mentioned

Meta-regression Number of obs = 18

REML estimate of between-study variance tau2 = .9547

% residual variation due to heterogeneity I-squared_res = 90.24%

Proportion of between-study variance explained Adj R-squared = 40.38%

Joint test for all covariates Model F(3,14) = 4.37

With Knapp-Hartung modification Prob > F = 0.0227

| _ES | Coefficient | Std. err. | t | P>\|t\| | [95% conf. interval] | |
| --- | --- | --- | --- | --- | --- | --- |
| Method of acupuncture | -.0365794 | .3838867 | -0.10 | 0.925 | -.8599345 | .7867757 |
| Needle retention time | .8554381 | .3623035 | 2.36 | 0.033 | .0783745 | 1.632502 |
| Duration | -1.337167 | .4915722 | -2.72 | 0.017 | -2.391485 | -.2828496 |
| _cons | -1.819305 | 1.261501 | -1.44 | 0.171 | -4.524955 | .8863445 |

**Method of acupuncture** is divided into three levels：

1、warm acupuncture; 2、electroacupuncture; 3、others;

**
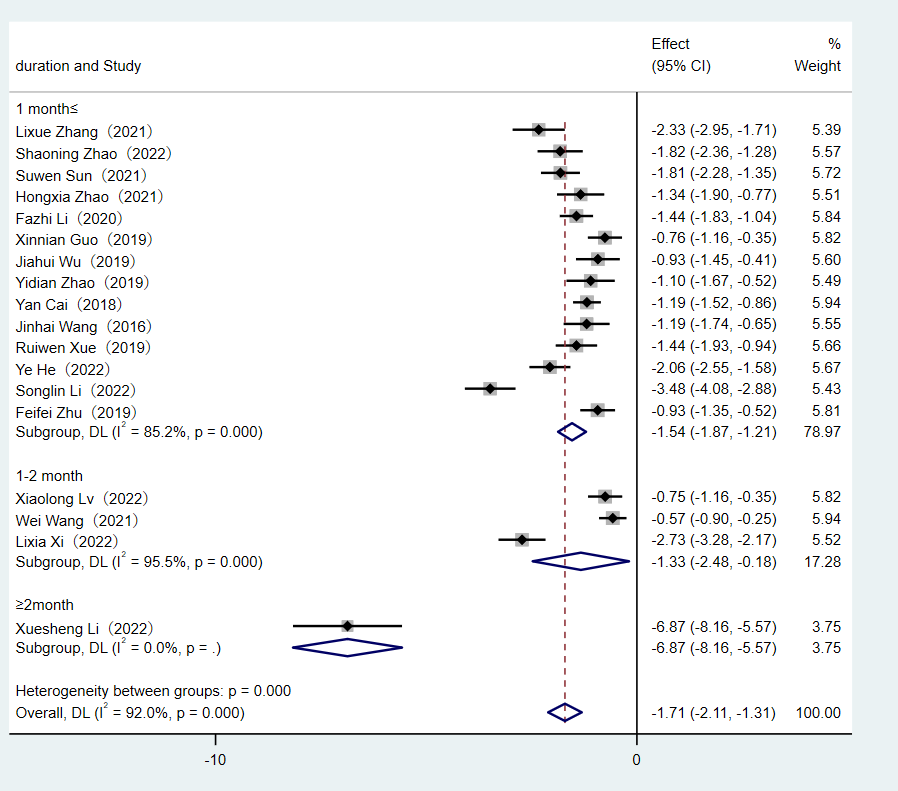
**

**Forest plot of TNF-α(Subgroup according to duration).**


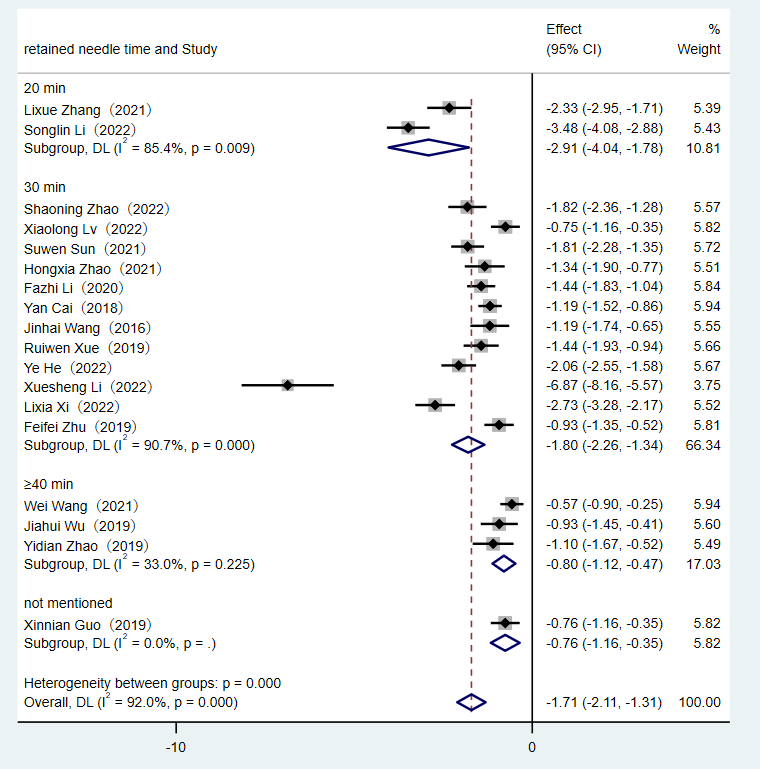


**Forest plot of TNF-α(Subgroup according to needle retention time).**

**IL-6**

**Duration** is divided into four levels：

1、7d≤；2、8-15d 3、16-30d 4、>1month

**Needle retention time** is divided into four levels：

1、20 min；2、30 min 3、≥40 min 4、no mentioned

Meta-regression Number of obs = 17

REML estimate of between-study variance tau2 = 1.928

% residual variation due to heterogeneity I-squared_res = 93.47%

Proportion of between-study variance explained Adj R-squared = 50.00%

Joint test for all covariates Model F(3,13) = 5.61

With Knapp-Hartung modification Prob > F = 0.0108

| _ES | Coefficient | Std. err. | t | P>\|t\| | [95% conf. interval] | |
| --- | --- | --- | --- | --- | --- | --- |
| Intervention | -3.851879 | 1.241915 | -3.10 | 0.008 | -6.534873 | -1.168886 |
| Duration | -.0052949 | .5138316 | -0.01 | 0.992 | -1.115361 | 1.104771 |
| Needle retained time | .7608274 | .4005648 | 1.90 | 0.080 | -.1045402 | 1.626195 |
| _cons | .7013852 | 1.850686 | 0.38 | 0.711 | -3.296778 | 4.699548 |

**Intervention** is divided into three levels：

1. conventional western treatment; 2、only used Rosuvastatin Calcium Tablets; 3、regular rehabilitation training;


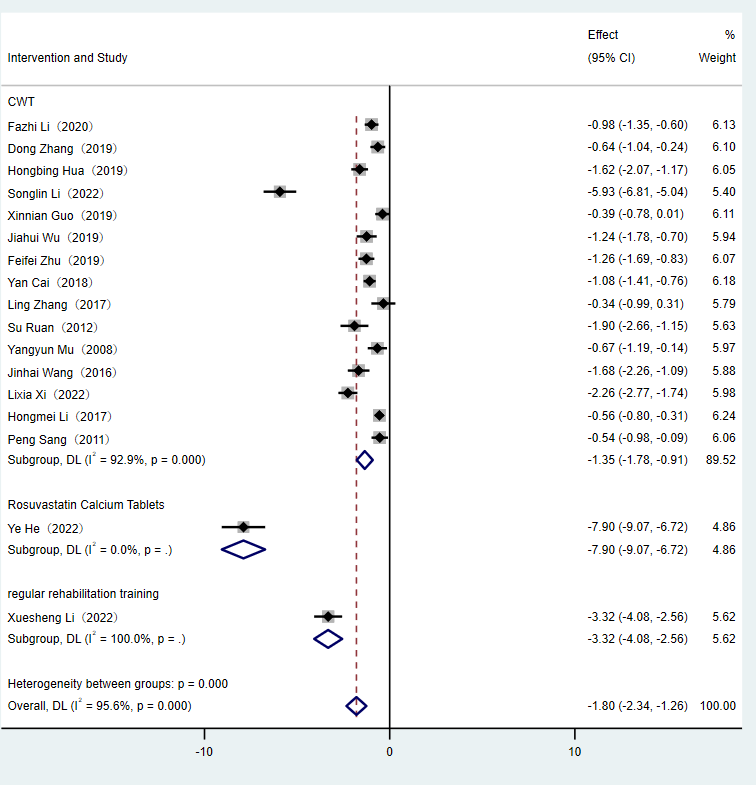


**Forest plot of IL-6 (Subgroup according to Intervention).**

**Hs-CRP**

**Duration** is divided into four levels：

1、7d≤；2、8-15d 3、16-30d 4、>1month

**The sample size** is divided into three levels：

1、100<; 2、100-149; 3、≥150

**The average of age** is divided into three levels：

1、60<; 2、60-70; 3、not mentioned

Meta-regression Number of obs = 17

REML estimate of between-study variance tau2 = .505

% residual variation due to heterogeneity I-squared_res = 89.23%

Proportion of between-study variance explained Adj R-squared = 31.21%

Joint test for all covariates Model F(3,13) = 3.19

With Knapp-Hartung modification Prob > F = 0.0596

| _ES | Coefficient | Std. err. | t | P>\|t\| | [95% conf. interval] | |
| --- | --- | --- | --- | --- | --- | --- |
| Age | -.0372735 | .3217668 | -0.12 | 0.910 | -.7324084 | .6578614 |
| The sample size | -.770093 | .2701405 | -2.85 | 0.014 | -1.353696 | -.18649 |
| Duration | .4976336 | .254803 | 1.95 | 0.073 | -.0528347 | 1.048102 |
| _cons | -1.103358 | .8321572 | -1.33 | 0.208 | -2.901124 | .6944085 |


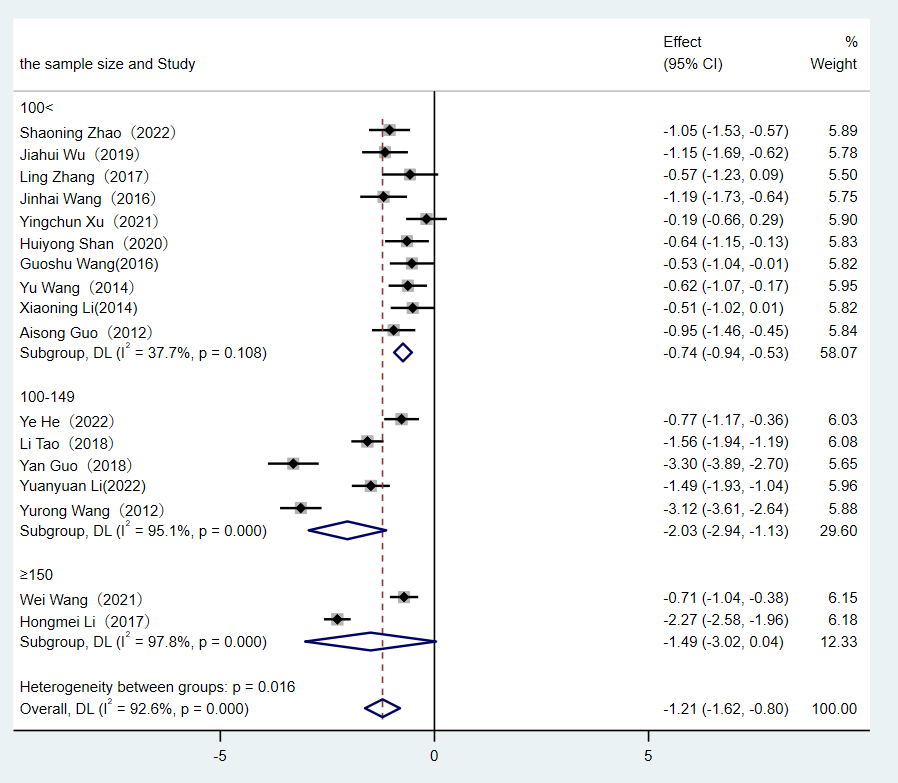


**Forest plot of hs-CRP(Subgroup according to the sample size).**

# Supplementary File S3. Lists the characteristics of included RCTs.

| \|  \|  \|  \|  \| **The average course of disease** \| \| **The number of case** \| \| **the average of age** \| \| **Intervention treatment** \| \|  \|  \|  \|  \| \| --- \| --- \| --- \| --- \| --- \| --- \| --- \| --- \| --- \| --- \| --- \| --- \| --- \| --- \| --- \| --- \| \| **Study** \| **Diseases** \| **Study design** \| **Diagnosis Criterion** \| **C** \| **E** \| **C** \| **E** \| **C** \| **E** \| **Control** \| **Acupuncture** \| **Usage of acupuncture** \| **Needle retention time** \| **Period of treatment (d)** \| **outcome** \| \| Shaoning Zhao（2022） \| ACI \| RTN \| o \| 6.50±1.25h \| 6.45±1.22h \| 38 \| 38 \| 62.55±10.18 \| 62.80±10.22 \| CWT \| CWT+electroacupuncture \| 1 time/d \| 30min \| 14 \| ①Ⅱ④Ⅱ⑥⑦ \| \| Ye He（2022） \| CI \| RTN \| g \| 10.04±2.79w \| 9.96±2.53w \| 50 \| 50 \| 59.31±6.28 \| 58.79±5.10 \| Rosuvastatin Calcium Tablets \| Rosuvastatin Calcium Tablets+Warm acupuncture \| 1 time/d, 5 time a week \| 30min \| 28 \| ①Ⅱ②Ⅲ⑥ \| \| Xiaolong Lv（2022） \| IS and spastic hemiplegia \| RTN \| NR \| 11.52 ± 3.23 d \| 11.38±3.18d \| 48 \| 52 \| 62.15 ±4.33 \| 61. 86 ±4. 45 \| aspirin+RRT \| aspirin+RRT+acupuncture \| NR \| 30min \| 2m \| ①Ⅱ⑤⑧ \| \| Xuesheng Li（2022） \| CI convalescence \| Random \| NR \| 3.28±0.44w \| 3.25±0.39w \| 32 \| 33 \| 54.98±7.74 \| 55.01±7.78 \| RRT \| RRT+acupuncture \| 1 time a week \| 30min \| 12w \| ①Ⅱ②Ⅲ③Ⅴ⑦ \| \| Songlin Li（2022） \| ACI \| Random，class stratum sampling \| g \| 3. 15 ± 0. 49h \| 3. 12 ± 0. 45h \| 54 \| 54 \| 56. 29 ± 6. 40 \| 56. 25 ± 6. 32 \| CWT+Citicoline and alteplase \| CWT+Citicoline and alteplase+acupuncture \| 1 time/d, 6 time a week \| 20min \| 28 \| ①Ⅲ②Ⅲ③Ⅲ⑤ \| \| Lixue Zhang（2021） \| ACI \| RTN \| g \| 3.56±0.62h \| 3.63±0.57h \| 34 \| 34 \| 59±8 \| 59±8 \| Interventional Thrombolysis in Cerebral Artery+hyperbaric \| Interventional Thrombolysis in Cerebral Artery+hyperbaric+acupuncture \| 1 time/d \| 20min \| 14 \| ①Ⅱ④Ⅱ⑤⑦⑧ \| \| Suwen Sun（2021） \| ACI \| RTN \| o \| 17.32± 2.65h \| 17.37± 2.64h \| 51 \| 51 \| 57.86±4.25 \| 57.81±4.29 \| CWT（Butylphthalide） \| CWT（Butylphthalide）+acupuncture \| 1 time/d \| 30min \| 28 \| ①Ⅱ⑤⑧ \| \| Wei Wang（2021） \| ACI \| RTN \| b \| 7.1±1.5w \| 6.9±1.4w \| 75 \| 75 \| 66.8±8.0 \| 67.0±6.9 \| CWT+RRT \| CWT+RRT+acupuncture \| 1 time/d, 5 time a week \| 60min \| 8w \| ①Ⅰ⑥ \| \| Hongxia Zhao（2021） \| CI \| Random \| g \| 17.567±4.191ｄ \| 17.800±3.906ｄ \| 30 \| 30 \| 63.933±３.095 \| 63.733±2.572 \| RRT \| RRT+acupunture abdominal \| 1 time/d, 5 time a week \| 30min \| 14 \| ①NR \| \| Daokuan Xia（2020） \| CI convalescence \| RTN \| b \| 6.13±1.45w \| 6.50±1.73w \| 45 \| 45 \| 57.18±6.48 \| 56.61±6.29 \| CWT \| CWT+Warm acupuncture \| 1 time/d, 5 time a week \| 30min \| 28 \| ④Ⅱ⑤⑦⑧ \| \| Fazhi Li（2020） \| CI \| RTN \| o \| 7.31±0.58d \| 7.28±0.64d \| 62 \| 62 \| 64.48±5.62 \| 65.17±5.76 \| CWT \| CWT+acupuncture \| 1 time/2d \| 30min \| 30 \| ①Ⅲ②Ⅱ⑤ \| \| Cunlei Jiang（2020） \| IS after CPR \| RTN \| o \| NR \| NR \| 20 \| 20 \| 72．35±13．90 \| 68．55±15．68 \| CWT \| CWT+acupuncture \| 1 time/d \| No needle retained \| 7 \| ③Ⅱ \| \| Hongbing Hua（2019） \| ACI \| RTN \| g \| NR \| NR \| 50 \| 50 \| 62±12 \| 64±9 \| CWT \| CWT+acupuncture \| 1 time/d \| 30min \| 14 \| ②Ⅲ④Ⅱ⑤⑧ \| \| Xinnian Guo（2019） \| ACI \| RTN \| b \| NR \| NR \| 50 \| 50 \| NR \| NR \| CWT \| CWT+acupuncture \| 1 time/d \| NR \| 28 \| ①Ⅲ②Ⅱ③Ⅳ \| \| Jiahui Wu（2019） \| ACI \| RTN \| g \| NR \| NR \| 31 \| 32 \| 66.37+ 3.62 \| 65.92±3.79 \| CWT+RRT \| CWT+RRT+acupuncture \| 1 time/d \| 40 min (20 am and 20 pm) \| 28 \| ①Ⅳ②Ⅱ⑤⑥⑦ \| \| Feifei Zhu（2019） \| CI in elderly \| RTN \| d \| NR \| NR \| 48 \| 52 \| 71±2 \| 71±2 \| CWT+Edaravone injection \| CWT+Edaravone injection+Warm acupuncture \| 1 time/d \| 30min \| 14 \| ①Ⅱ②Ⅱ③Ⅱ⑤⑦⑧ \| \| Yidian Zhao（2019） \| IS \| RTN \| g \| 7 ～ 14 d \| \| 27 \| 27 \| NR \| NR \| RRT \| RRT+acupuncture \| 5-6 time a week \| 7-8h \| 14 \| ①Ⅲ \| \| Yan Cai（2018） \| ACI \| RTN \| d \| 2.85±1.26d \| 2.73±1.30d \| 83 \| 83 \| 63.09±8.84 \| 62.92±9.71 \| CWT+RRT \| CWT+RRT+electronacupuncture \| 1 time/d \| 30min \| 28 \| ①Ⅱ②Ⅱ③Ⅲ⑦ \| \| Ling Zhang（2017） \| ACI \| RTN \| d \| NR \| NR \| 19 \| 18 \| 66．53±8．79 \| 64．89± 9．42 \| CWT \| CWT+acupuncture \| 1 time/d, 6 time a week \| 30min \| 28 \| ②Ⅰ⑤⑥⑦ \| \| Su Ruan（2012） \| ACI \| Random \| d \| NR \| NR \| 20 \| 20 \| 60.11±8.01 \| 62.20±9.05 \| CWT \| CWT+scalpel \| 1 time/d \| 20min \| 14 \| ②Ⅱ⑤ \| \| Yangyun Mu（2008） \| ACI \| Random，single-blind \| d \| 2.18±0.96d \| 2.11±1.09d \| 30 \| 30 \| 66．5±8．81 \| 69．5±7．10 \| CWT \| CWT+scalpel \| NR \| No needle retained \| 20 \| ②Ⅱ \| \| Jinhai Wang（2016） \| ACI \| Stratified block randomization \| d \| 13.7±7.7ｈ \| 13.0±7.7ｈ \| 30 \| 31 \| 55.8±9.9 \| 54.9±10.7 \| CWT \| CWT+head acupuncture \| 1 time/d \| 30 min \| 7 \| ①Ⅲ②Ⅲ④Ⅱ⑤⑥ \| \| Lixia Xi（2022） \| CI \| RTN \| g \| ≤ 30 d \| \| 48 \| 48 \| 45 ~ 75 \| \| CWT+RRT \| CWT+RRT+acupuncture \| 1 time/d, 5 time a week \| 30min \| 8w \| ①Ⅱ②Ⅱ⑦ \| \| Yingchun Xu（2021） \| ACI \| RTN \| NR \| １０.６５±２.５７d \| １１.２６±２.２９d \| 34 \| 34 \| 59.65±9.90 \| 59.68±7.81 \| CWT+Hyperbaric oxygen therapy \| CWT+Hyperbaric oxygen therapy+acupuncture \| 1 time/d \| 90min \| 10 \| ⑥⑦⑧ \| \| Huiyong Shan（2020） \| ACI \| RTN \| g \| ＜48h \| \| 31 \| 31 \| 61.85±6.96 \| 60.99±6.79 \| CWT \| CWT+acupuncture \| 1 time/d \| No needle retained \| 21 \| ⑤⑥ \| \| Dong Zhang（2019） \| CI \| Random \| d \| 16.21±2.12y \| 15.39±2.73y \| 50 \| 50 \| 65.23±10.75 \| 64.50±7.42 \| CWT \| CWT+acupuncture \| NR \| 30min \| 28 \| ②Ⅱ⑤ \| \| Ruiwen Xue（2019） \| IS \| RTN \| o \| 8．12±0．39h \| 8．01±0．43h \| 40 \| 40 \| 59.26±7.63 \| 60.14±7.82 \| CWT \| CWT+acupuncture \| 1 time a week \| 30min \| 28 \| ①Ⅱ④Ⅱ⑤⑦ \| \| Li Tao（2018） \| ACI \| RTN \| b \| 17.24±8.23 h \| 17.87±9.05h \| 71 \| 71 \| 65.03±6.82 \| 64.67±6.31 \| CWT \| CWT+acupuncture \| 1 time/d \| No needle retained \| 14 \| ⑤⑥⑦ \| \| Yan Guo（2018） \| ACI \| RTN \| d \| ＜24h \| \| 52 \| 52 \| 60.97±1.98 \| 61.05±2.15 \| CWT \| CWT+acupuncture \| 1 time/d \| 30min \| 7 \| ⑥⑦ \| \| Jun Xiang（2018） \| CI \| RTN \| g \| NR \| NR \| 60 \| 60 \| 55.06 ±0.35 \| 55.04±0.34 \| CWT+RRT \| CWT+RRT+acupuncture \| 1 time/d, 5 time a week \| 30min \| 14 \| ③Ⅱ⑤⑦ \| \| Hai Tao Wang（2017） \| CI \| Random \| d \| 33.82±3.97h \| 34.02±4.02h \| 32 \| 32 \| 56±7 \| 56±7 \| Edaravone and plasmin injection \| Edaravone and plasmin injection+acupuncture \| 1 time/d, 5 time a week \| No needle retained \| 14 \| ③Ⅱ⑤⑧ \| \| Hongmei Li（2017） \| IS \| Random，toss a coin \| g \| 6.03±1.21ｈ \| 5.94±1.27ｈ \| 130 \| 130 \| 62.72±5.84 \| 62.82±5.75 \| CWT \| CWT+acupuncture \| 1 time/d \| 30min \| 14 \| ①Ⅱ②Ⅱ⑥⑦ \| \| Xiaoli Liu（2016） \| ACI \| RTN \| d \| 32.23± 12.57h \| 31 ± 11. 32 h \| 33 \| 33 \| 55． 45 ± 6． 03 \| 54． 15 ± 6． 87 \| Edaravone and plasmin injection \| Edaravone and plasmin injection+acupuncture \| 1 time/d, 5 time a week \| 30min \| 14 \| ⑧ \| \| Guoshu Wang(2016) \| CI \| Random \| d \| 8.98±5.39d \| 10.52±4.74d \| 30 \| 30 \| 57±6 \| 53±8 \| RRT \| RRT+acupuncture \| 1 time/d \| 45min \| 10 \| ⑥ \| \| Xiaowei Sun（2015） \| ACI \| RTN \| NR \| NR \| NR \| 51 \| 51 \| 58.63 ±8.67 \| 57.67 ±9.21 \| CWT \| CWT+acupuncture \| 1 time/d \| 50min \| 28 \| ⑤ \| \| Wenlin Yu（2015） \| CI \| Random \| d \| 2.3±1.1month \| 2.1±0.9 month \| 30 \| 30 \| 60.1±3.5 \| 59.6±2.9 \| RRT \| RRT+acupuncture \| 1 time/d \| 30min \| 20 \| ③Ⅱ \| \| Yu Wang（2014） \| ACI \| RTN \| d \| ＜7d \| \| 40 \| 40 \| 60±8 \| 60±9 \| CWT \| CWT+acupuncture \| 1 time/d \| 60min \| 14 \| ⑥⑦ \| \| Xiaoning Li(2014) \| ACI \| Random \| d \| NR \| NR \| 30 \| 30 \| 62±11 \| 62±11 \| CWT \| CWT+acupuncture \| 1 time/d \| 6h \| 14 \| ⑥ \| \| Yuanyuan Li(2022) \| ACI \| Random \| NR \| 1.45±0.55d \| 1.53±0.47d \| 50 \| 50 \| 59.75 ±4.25 \| 58.65 ±4.35 \| CWT \| CWT+acupuncture \| 1 time/d \| 30min \| 14 \| ⑤⑥⑦⑧ \| \| Peng Sang（2013） \| ACI \| RTN \| d \| NR \| NR \| 40 \| 40 \| 59.65±8.34 \| \| CWT \| CWT+acupuncture \| 1 time/d \| 30min \| 14 \| ⑤ \| \| Aisong Guo（2012） \| ACI \| RTN \| d \| ＜3d \| \| 33 \| 34 \| 41～73 \| 42～75 \| RRT \| RRT+acupuncture \| 1 time/d \| 30min \| 14 \| ⑥⑦⑧ \| \| Yurong Wang（2012） \| ACI \| RTN \| d \| 7.1± 2.3h \| 7.1± 2.8h \| 73 \| 73 \| 54． 7 ± 10． 1 \| 55． 1 ± 12． 3 \| CWT \| CWT+acupuncture \| 1 time/d, 5 time a week \| 30min \| 21 \| ⑥ \| \| Peng Sang（2011） \| ACI \| RTN \| NR \| NR \| NR \| 40 \| 40 \| NR \| NR \| CWT \| CWT+acupuncture \| NR \| NR \| 14 \| ①Ⅵ②Ⅲ \| |
| --- | --- | --- | --- | --- | --- | --- | --- | --- | --- | --- | --- | --- | --- | --- | --- | --- | --- | --- | --- | --- | --- | --- | --- | --- | --- | --- | --- | --- | --- | --- | --- | --- | --- | --- | --- | --- | --- | --- | --- | --- | --- | --- | --- | --- | --- | --- | --- | --- | --- | --- | --- | --- | --- | --- | --- | --- | --- | --- | --- | --- | --- | --- | --- | --- | --- | --- | --- | --- | --- | --- | --- | --- | --- | --- | --- | --- | --- | --- | --- | --- | --- | --- | --- | --- | --- | --- | --- | --- | --- | --- | --- | --- | --- | --- | --- | --- | --- | --- | --- | --- | --- | --- | --- | --- | --- | --- | --- | --- | --- | --- | --- | --- | --- | --- | --- | --- | --- | --- | --- | --- | --- | --- | --- | --- | --- | --- | --- | --- | --- | --- | --- | --- | --- | --- | --- | --- | --- | --- | --- | --- | --- | --- | --- | --- | --- | --- | --- | --- | --- | --- | --- | --- | --- | --- | --- | --- | --- | --- | --- | --- | --- | --- | --- | --- | --- | --- | --- | --- | --- | --- | --- | --- | --- | --- | --- | --- | --- | --- | --- | --- | --- | --- | --- | --- | --- | --- | --- | --- | --- | --- | --- | --- | --- | --- | --- | --- | --- | --- | --- | --- | --- | --- | --- | --- | --- | --- | --- | --- | --- | --- | --- | --- | --- | --- | --- | --- | --- | --- | --- | --- | --- | --- | --- | --- | --- | --- | --- | --- | --- | --- | --- | --- | --- | --- | --- | --- | --- | --- | --- | --- | --- | --- | --- | --- | --- | --- | --- | --- | --- | --- | --- | --- | --- | --- | --- | --- | --- | --- | --- | --- | --- | --- | --- | --- | --- | --- | --- | --- | --- | --- | --- | --- | --- | --- | --- | --- | --- | --- | --- | --- | --- | --- | --- | --- | --- | --- | --- | --- | --- | --- | --- | --- | --- | --- | --- | --- | --- | --- | --- | --- | --- | --- | --- | --- | --- | --- | --- | --- | --- | --- | --- | --- | --- | --- | --- | --- | --- | --- | --- | --- | --- | --- | --- | --- | --- | --- | --- | --- | --- | --- | --- | --- | --- | --- | --- | --- | --- | --- | --- | --- | --- | --- | --- | --- | --- | --- | --- | --- | --- | --- | --- | --- | --- | --- | --- | --- | --- | --- | --- | --- | --- | --- | --- | --- | --- | --- | --- | --- | --- | --- | --- | --- | --- | --- | --- | --- | --- | --- | --- | --- | --- | --- | --- | --- | --- | --- | --- | --- | --- | --- | --- | --- | --- | --- | --- | --- | --- | --- | --- | --- | --- | --- | --- | --- | --- | --- | --- | --- | --- | --- | --- | --- | --- | --- | --- | --- | --- | --- | --- | --- | --- | --- | --- | --- | --- | --- | --- | --- | --- | --- | --- | --- | --- | --- | --- | --- | --- | --- | --- | --- | --- | --- | --- | --- | --- | --- | --- | --- | --- | --- | --- | --- | --- | --- | --- | --- | --- | --- | --- | --- | --- | --- | --- | --- | --- | --- | --- | --- | --- | --- | --- | --- | --- | --- | --- | --- | --- | --- | --- | --- | --- | --- | --- | --- | --- | --- | --- | --- | --- | --- | --- | --- | --- | --- | --- | --- | --- | --- | --- | --- | --- | --- | --- | --- | --- | --- | --- | --- | --- | --- | --- | --- | --- | --- | --- | --- | --- | --- | --- | --- | --- | --- | --- | --- | --- | --- | --- | --- | --- | --- | --- | --- | --- | --- | --- | --- | --- | --- | --- | --- | --- | --- | --- | --- | --- | --- | --- | --- | --- | --- | --- | --- | --- | --- | --- | --- | --- | --- | --- | --- | --- | --- | --- | --- | --- | --- | --- | --- | --- | --- | --- | --- | --- | --- | --- | --- | --- | --- | --- | --- | --- | --- | --- | --- | --- | --- | --- | --- | --- | --- | --- | --- | --- | --- | --- | --- | --- | --- | --- | --- | --- | --- | --- | --- | --- | --- | --- | --- | --- | --- | --- | --- | --- | --- | --- | --- | --- | --- | --- | --- | --- | --- | --- | --- | --- | --- | --- | --- | --- | --- | --- | --- | --- | --- | --- | --- | --- | --- | --- | --- | --- | --- | --- | --- | --- | --- | --- | --- | --- | --- | --- | --- | --- | --- | --- | --- | --- | --- | --- | --- | --- | --- | --- | --- | --- | --- | --- | --- | --- | --- | --- | --- | --- | --- | --- | --- | --- | --- | --- | --- | --- | --- | --- | --- | --- | --- | --- | --- | --- | --- | --- | --- | --- | --- | --- | --- | --- | --- | --- | --- | --- | --- | --- | --- | --- | --- | --- | --- | --- | --- | --- | --- | --- | --- | --- | --- | --- | --- | --- | --- |

Abbreviations: E: acupuncture group，C: Control group; RTN：Random number table method； ACI: Acute cerebral infarction; CI: cerebral infarction; CWT: conventional western treatment; RRT: regular rehabilitation training; d. Diagnosis of various types of cerebrovascular (1995, 1996 or 1998 version) published on The Fourth China Academic Conference on Cerebrovascular Diseases; g. Guidelines for the diagnosis and treatment of acute ischemic stroke in China (2010, 2013, 2014, 2017 or 2018 version) ; o.Other lectures except a related to CI diagnosis; b.Based on laboratory examination,symptoms and signs of CI; NR: Not report; ①Tumor necrosis factor-α(TNF-α); ②Interleukin-6 (IL-6); ③Interleukin-1β(IL-1β); ④C-reactive protein (CRP); ⑤Total Clinical Effective Rate(TCER); ⑥hypersensitive-c-reactive-protein(hs-CRP); ⑦NIHSS score;⑧adverse event(AE) rate; The unit of outcome measure：**Ⅰ**.pg/L; **Ⅱ**.pg/ml or ng/L; **Ⅲ**.ng/ml or ug/L; **Ⅳ**.ug/ml or mg/L; **Ⅴ**.mg/ml; **Ⅵ**.fmol/L

# Supplementary File S4 Results of the quality of evidence

| **Certainty assessment** | | | | | | | **Summary of findings** | | | **Comments** |
| --- | --- | --- | --- | --- | --- | --- | --- | --- | --- | --- |
| **Participants**  **(studies)**  **Follow-up** | **Risk of bias** | **Inconsistency** | **Indirectness** | **Imprecision** | **Other consideration** | **Overall certainty of evidence** | **Events** | | **Anticipated absolute effects or Relative effect**  **(95% CI)** |  |
|  |  |  |  |  |  |  | **Control** | **Experiment** |  |  |
| TNF-α  786 (9 RCTs) | serious^a^ | not serious | not serious | not serious | strong association | ⨁⨁⨁⨁  High | 392 | 394 | SMD 1.36 lower  （1.51 lower to 1.20 lower) | Risk of bias(-1^a^) |
| IL-6  1190(17 RCTs) | serious^a^ | serious^c^ | not serious | not serious | strong association | ⨁⨁⨁◯  Moderate | 593 | 597 | SMD 0.82 lower  （0.94 lower to 0.70 lower) | Risk of bias(-1^a^)  Inconsistency(-1^c^) |
| hs-CRP  1065 (13 RCTs) | serious^a^ | serious^c^ | not serious | not serious | strong association | ⨁⨁⨁◯  Moderate | 532 | 533 | SMD 0.86 lower  （0.99 lower to 0.74 lower) | Risk of bias(-1^a^)  Inconsistency(-1^c^) |
| Total effective rate  1843 (21 RCTs) | serious^a^ | not serious | not serious | not serious | Very strong association | ⨁⨁⨁⨁  High | 716/917  (78.1%) | 868/926  (93.7%) | RR 1.11  (1.03 to 1.19) | Risk of bias(-1^a^) |
| NIHSS scores  1782 (18 RCTs) | serious^a^ | very serious^d^ | serious^b^ | not serious | Very strong assoc.iation | ⨁⨁◯◯  Low | 888 | 894 | SMD 1.23 lower  （1.62 lower to 0.83 lower) | Risk of bias(-1^a^)  Inconsistency(-2^d^)  Indirectness(-1^b^) |
| CRP  823 (9 RCTs) | serious^a^ | very serious^d^ | not serious | not serious | Very strong association | ⨁⨁⨁◯  Moderate | 409 | 414 | SMD 3.16 lower  (4.08 lower to 2.24 lower) | Risk of bias(-1^a^)  Inconsistency(-2^d^) |
| IL-1β  285 (4 RCTs) | serious^a^ | serious^c^ | serious^f^ | serious^e^ | None | ⨁◯◯◯  Very low | 142 | 143 | SMD 1.10 lower  （1.35 lower to 0.85 lower) | Risk of bias(-1^a^)  Inconsistency(-1^c^)  Indirectness(-1^e^) |
| AE rate  1074 (11 RCTs) | serious^a^ | not serious | serious^g^ | not serious | strong association | ⨁⨁⨁◯  Moderate | 70/458 (15.3%) | 46/467 (9.9%) | RR 0.71  （0.49 to 1.01) | Risk of bias(-1^a^)  Indirectness(-1^g^) |

**Note:**  **RR:** risk ratio; **SMD:** Standardized mean difference. **Explanations:** a. There exists unclear risk of bias as showed in Table 2; b. 17 studies showed that the scores in acupuncture were smaller than conventional group，while 1 study was smaller;c. The direction of the effect is different as 50%＜I^2^＜75; d. The direction of the effect is different as 75%＜I^2^; e. the number of studies is small; f.The sample size was too small; g. 7 studies showed that the adverse events in acupuncture were smaller than conventional group，while 4 study was more.

# Supplementary File S5 Supplemental Figure


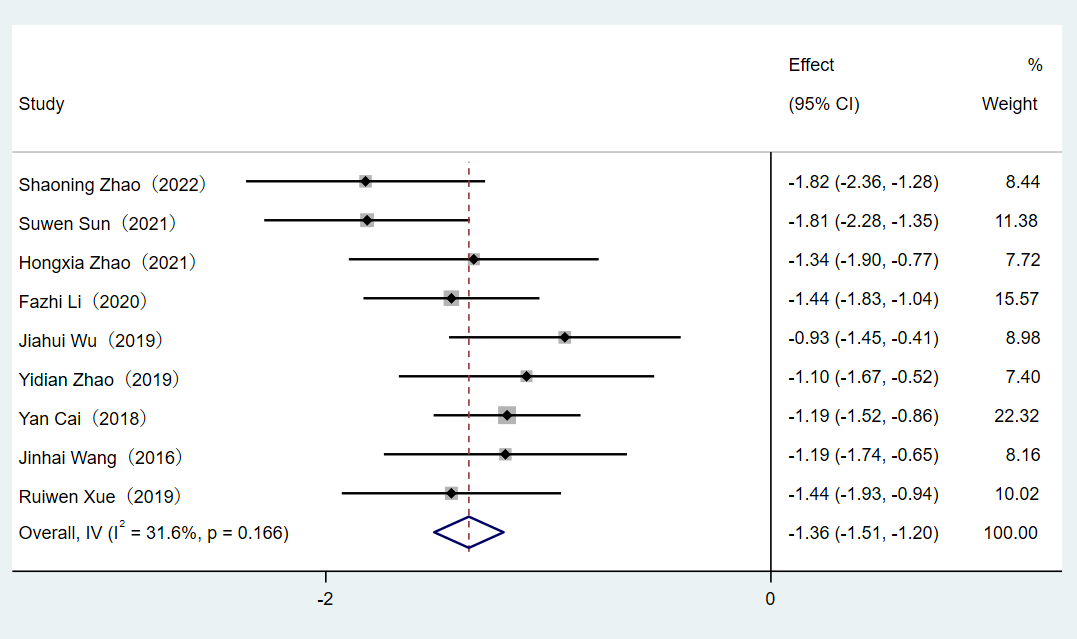


**Supplemental Fig 1 Forest plot of TNF-α after sensitivity analysis**

**
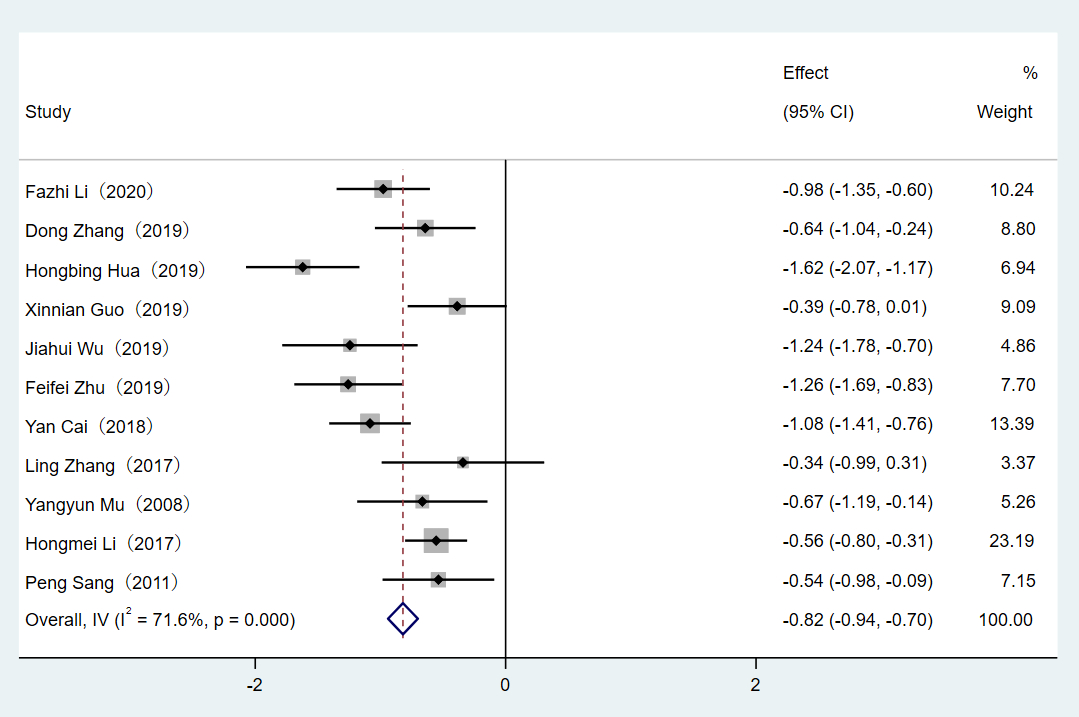
**

**Supplemental Fig 2 Forest plot of IL-6 after sensitivity analysis**

**
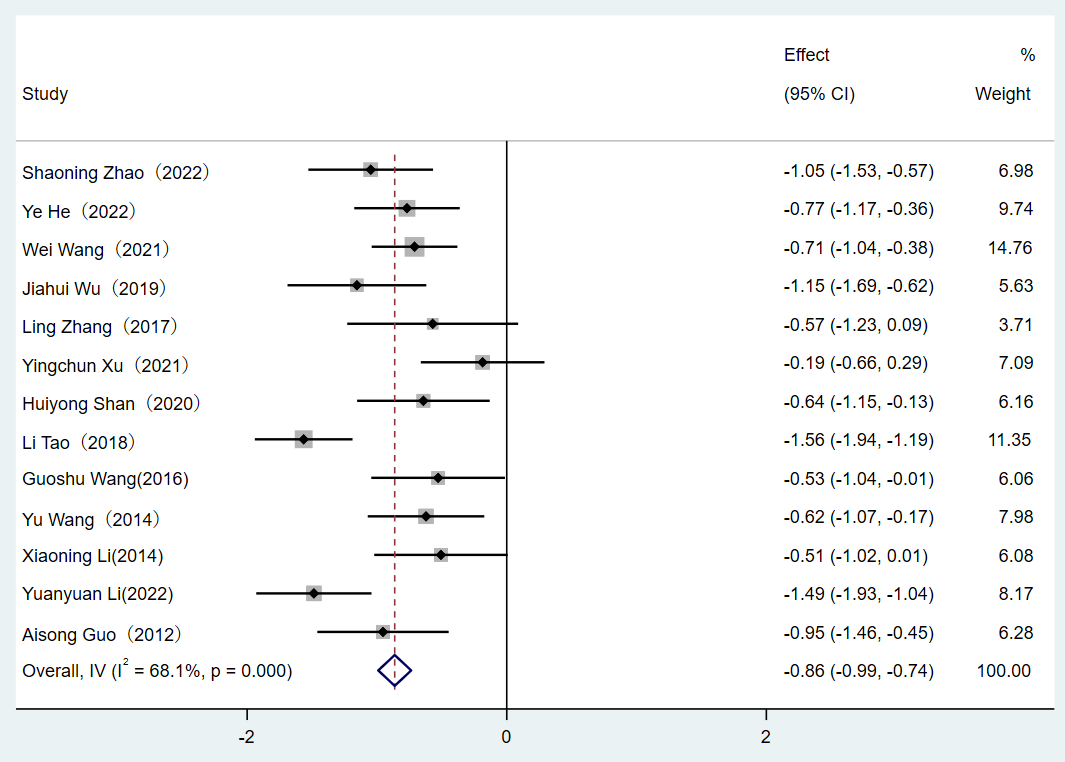
**

**Supplemental Fig 3 Forest plot of hs-CRP before sensitivity analysis**

**
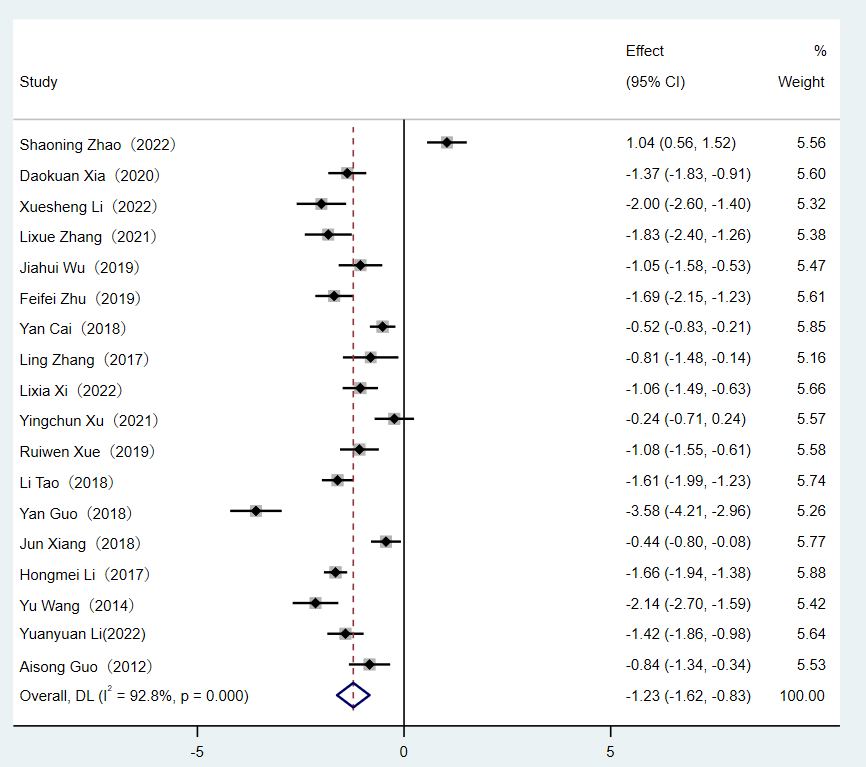
**

**Supplemental Fig 4 Forest plot of NIHSS before sensitivity analysis**

**
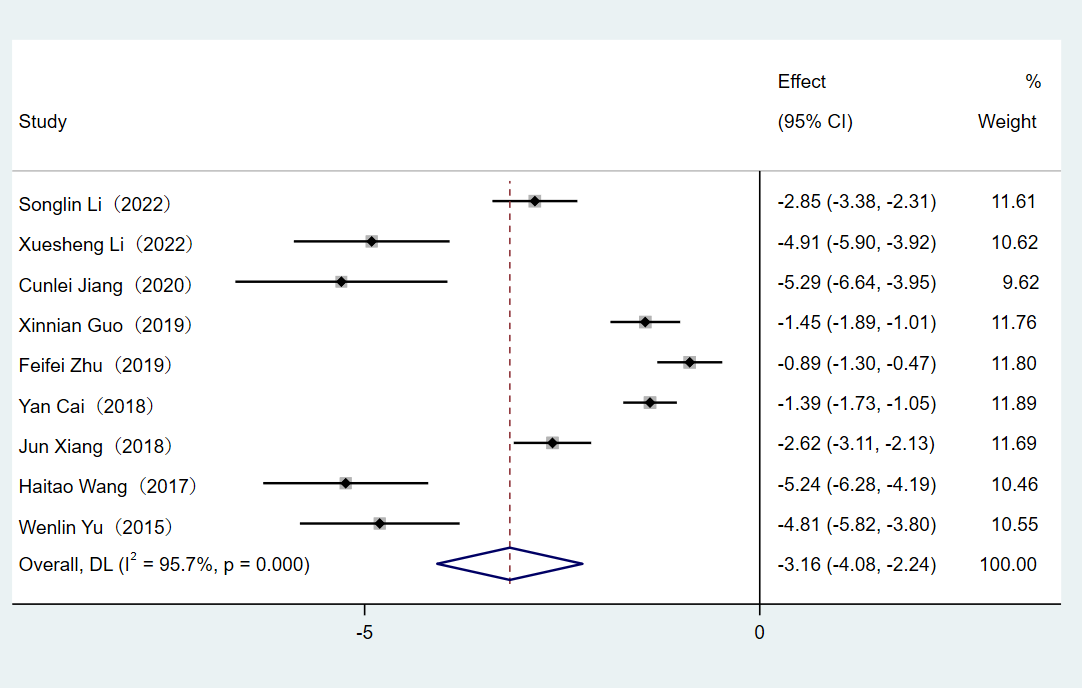
**

**Supplemental Fig 5 Forest plot of CRP before sensitivity analysis**

**
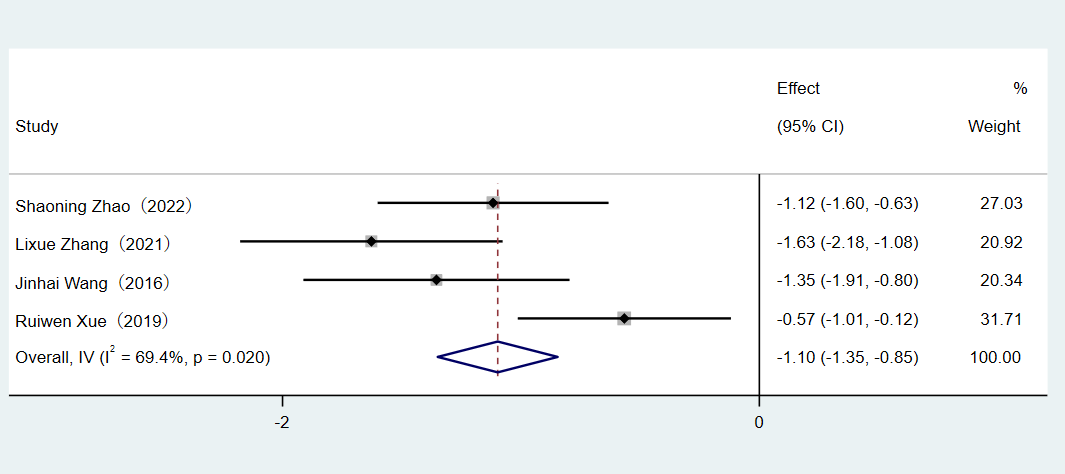
**

**Supplemental Fig 6 Forest plot of IL-1β before sensitivity analysis.**

# Supplementary File S6 Figure

**Figure 1 A PRISMA flow diagram of the literature screening and selection process.**
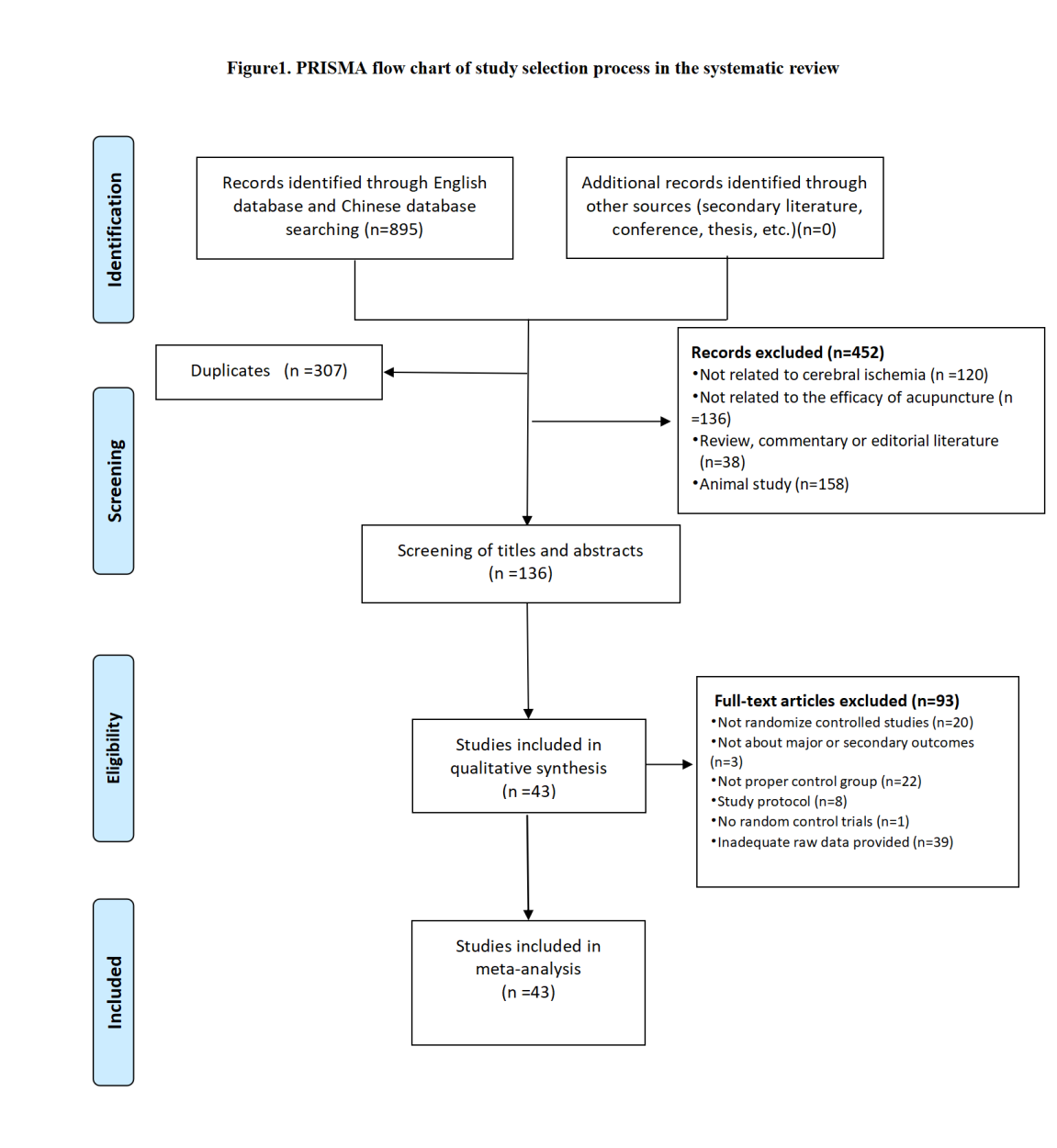


**Figure 2 Results of the risk of bias assessment using ROB2**
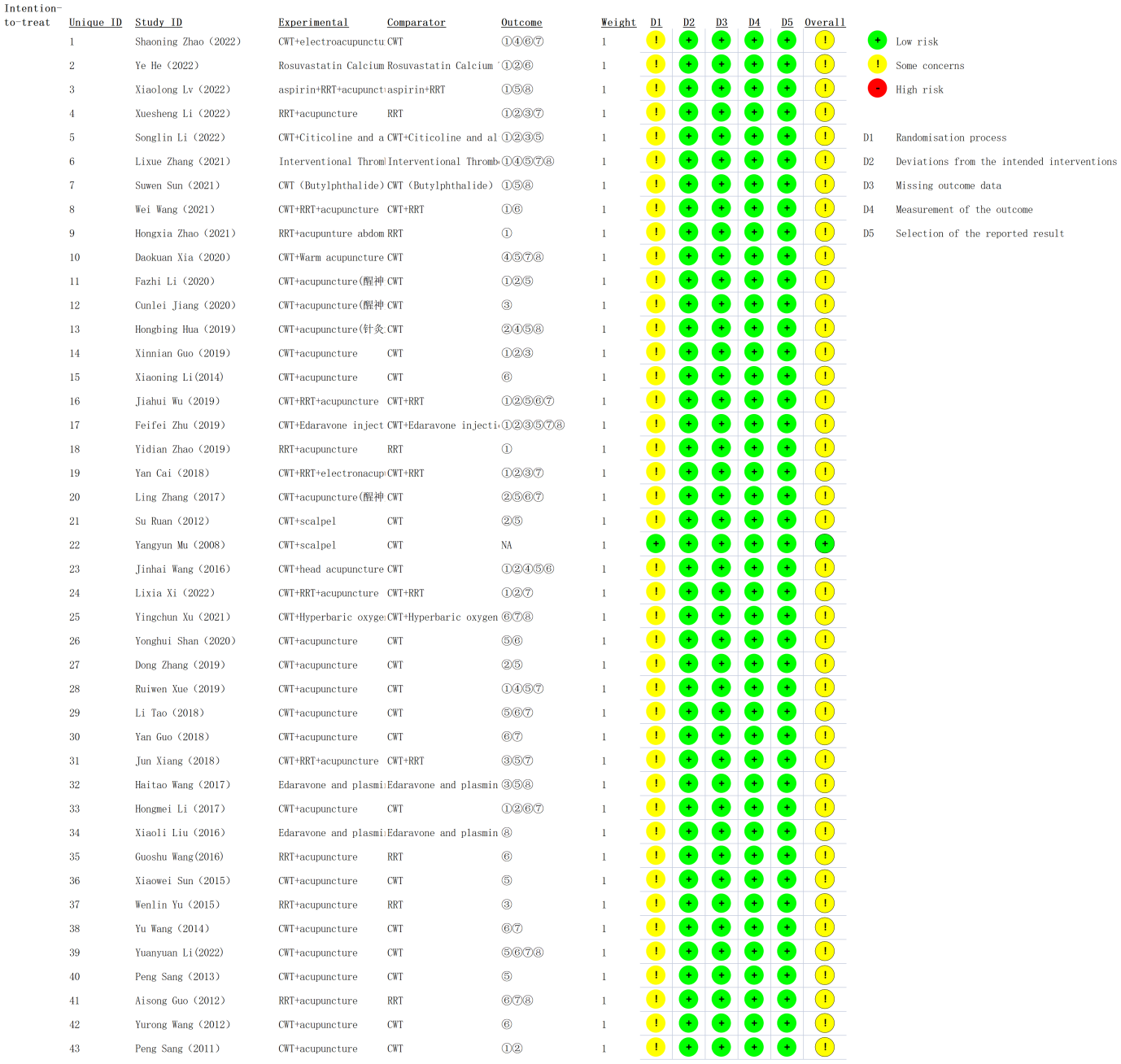


**Figure 3 The usage frequency of acupuncture points and medians. (A) The top 10 most frequency acupuncture points; (B) The frequency of each median; (C) The proportion of the 10 most frequency acupuncture points; (D) The proportion of the frequency of each median; (E) Network diagram of the combination rules in selected acupuncture points.**
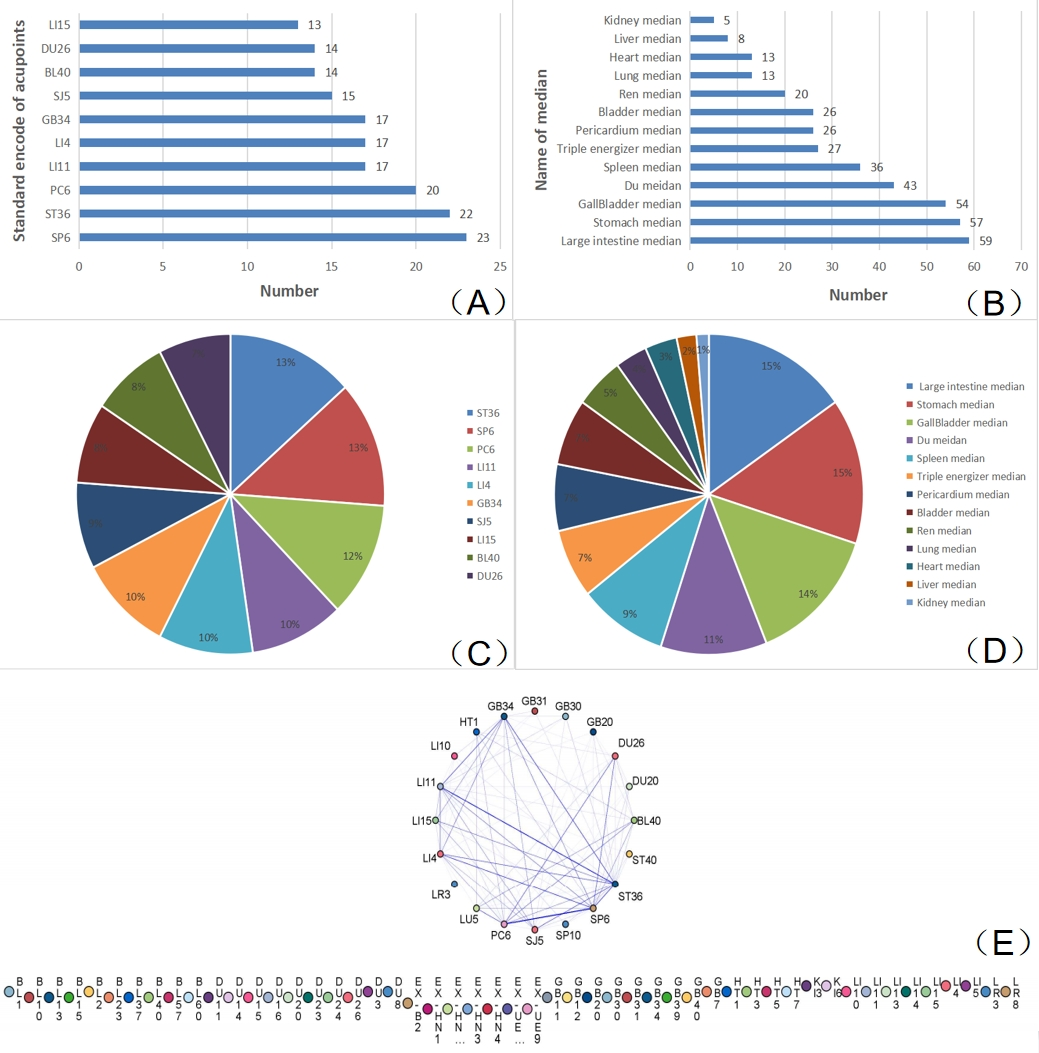


**Figure 4 Forest plot of TNF-α(Subgroup according to course of disease).**
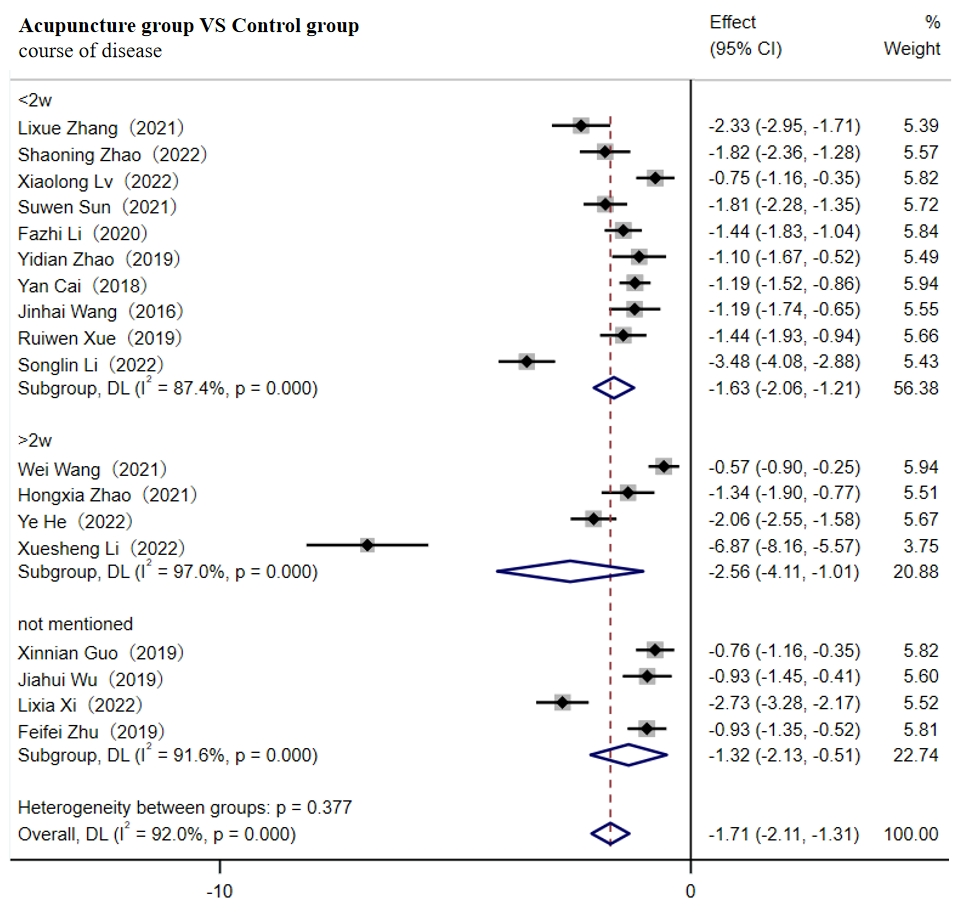


**Figure 5 Forest plot of IL-6(Subgroup according to course of disease).**
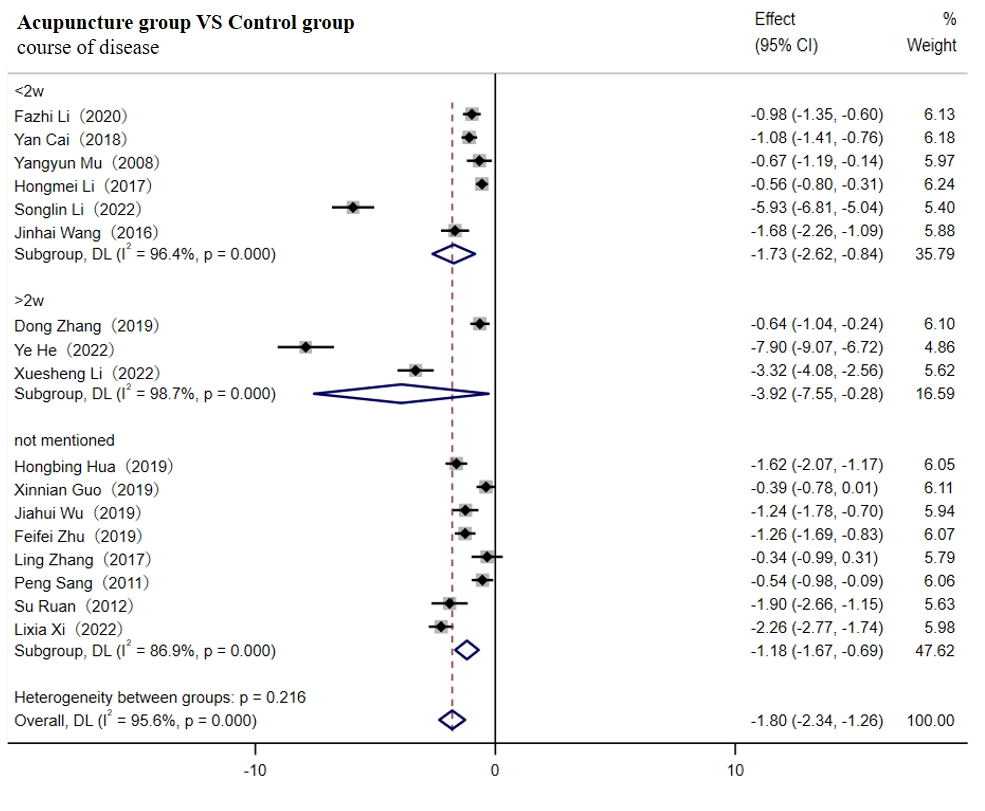


**Figure 6 Forest plot of hs-CRP (Subgroup according to course of disease).
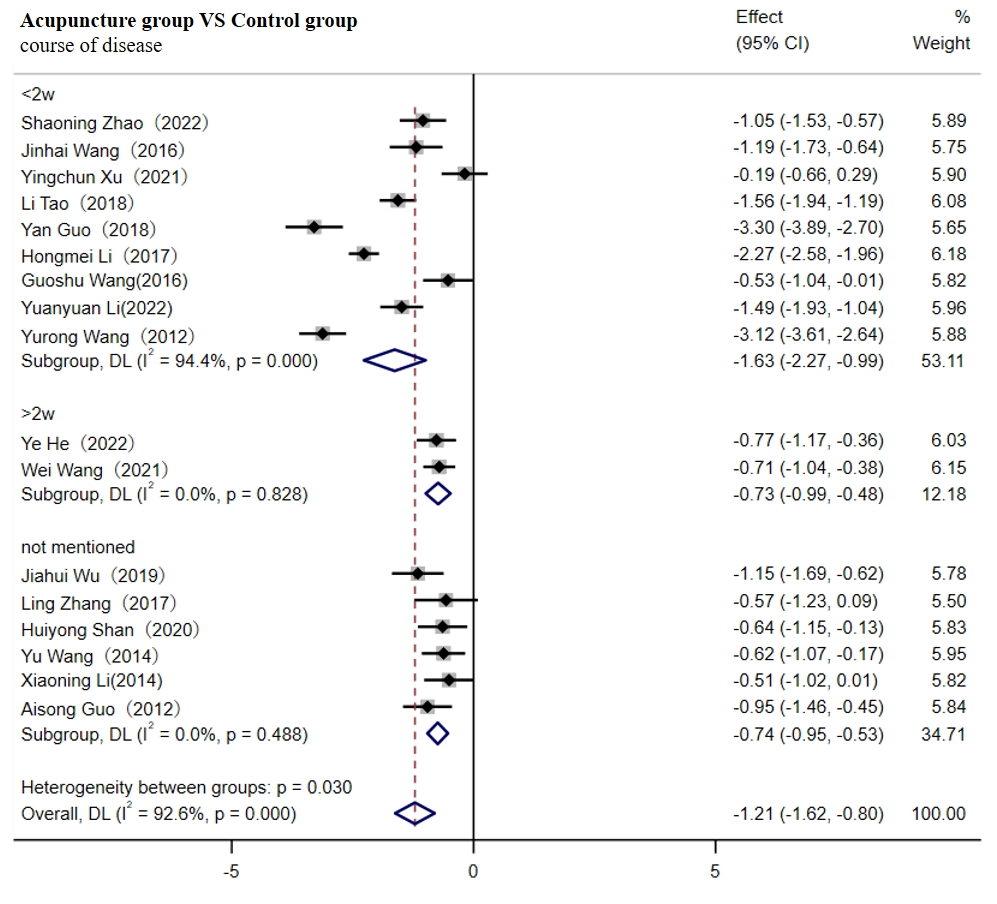
**

**Figure 7 Forest plot of TCER.
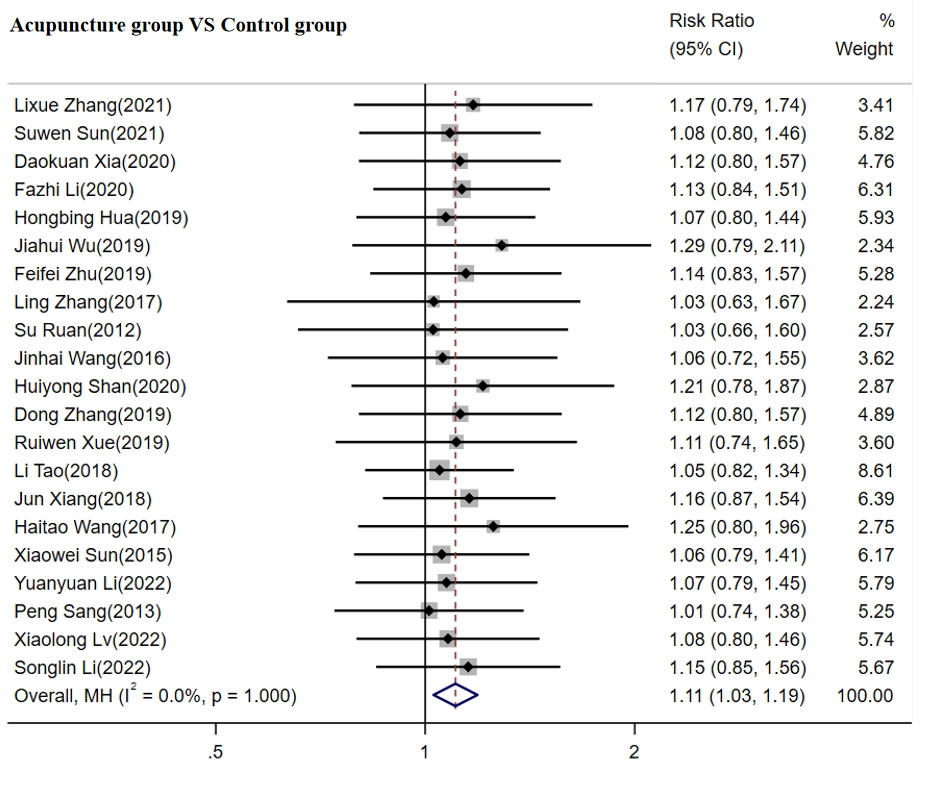
**

**Figure 8 Forest plot of NIHSS scores(Subgroup according to course of disease).**
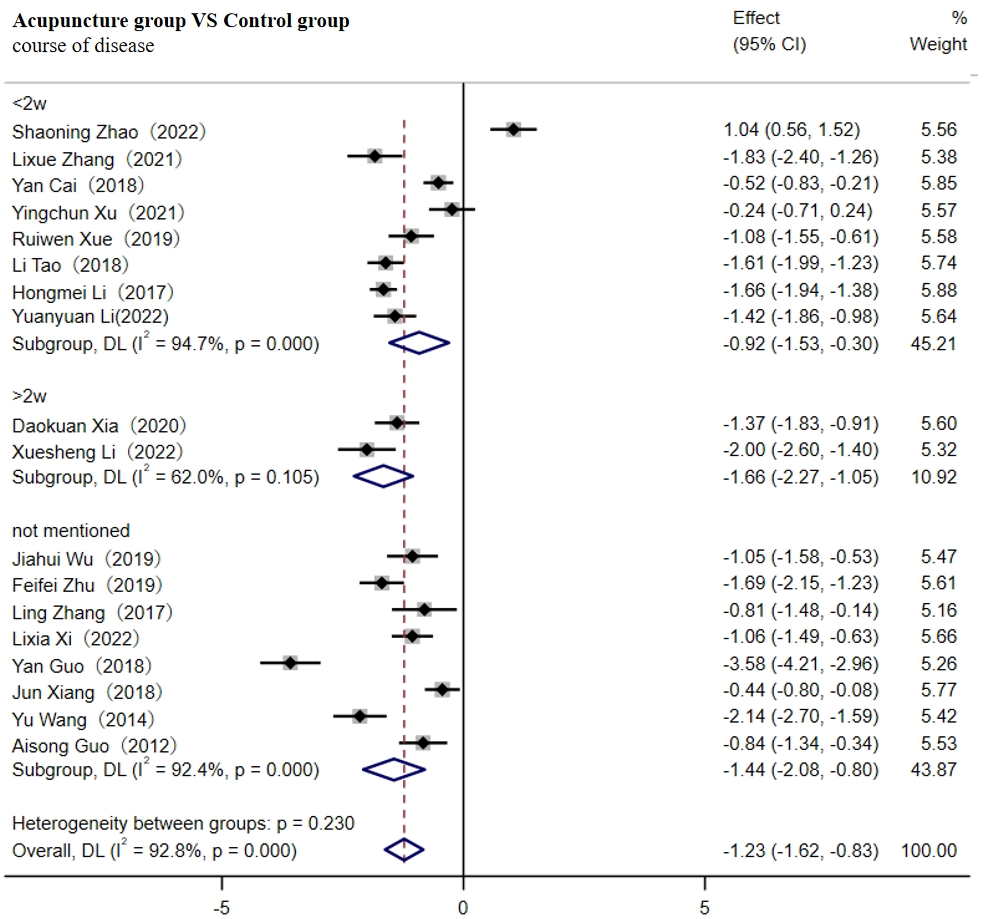


**Figure 9 Forest plot of CRP(Subgroup according to course of disease).
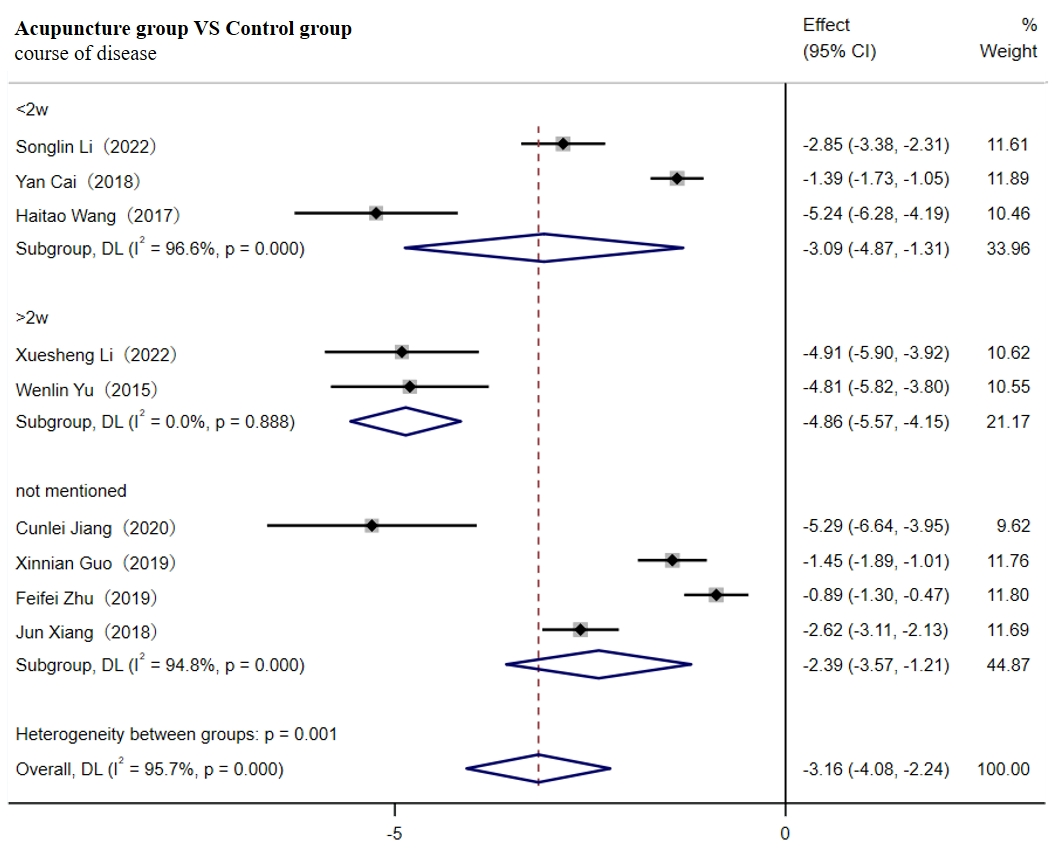
**

**Figure 10 Forest plot of IL-1β before sensitivity analysis.
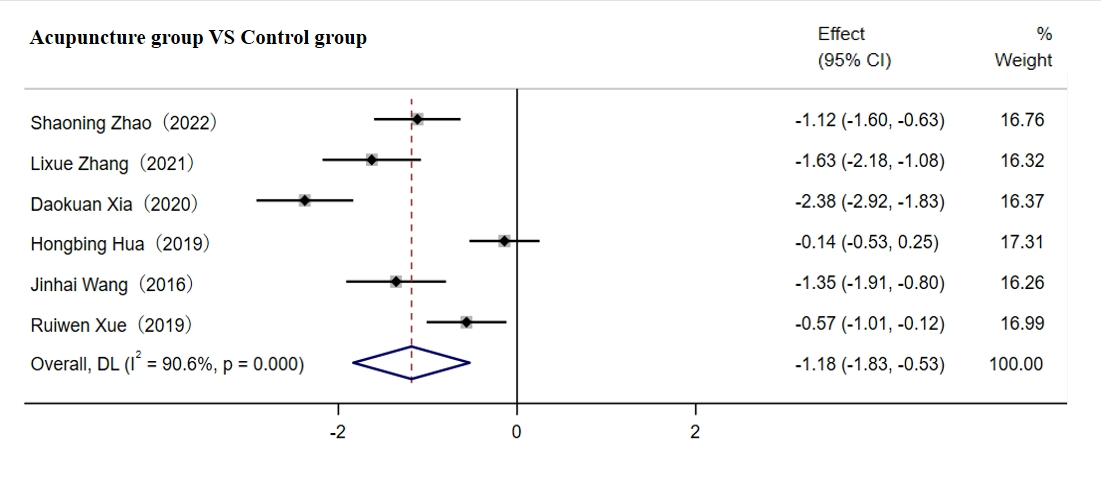
**

**Figure 11 Forest plot of Adverse Events comparison.**
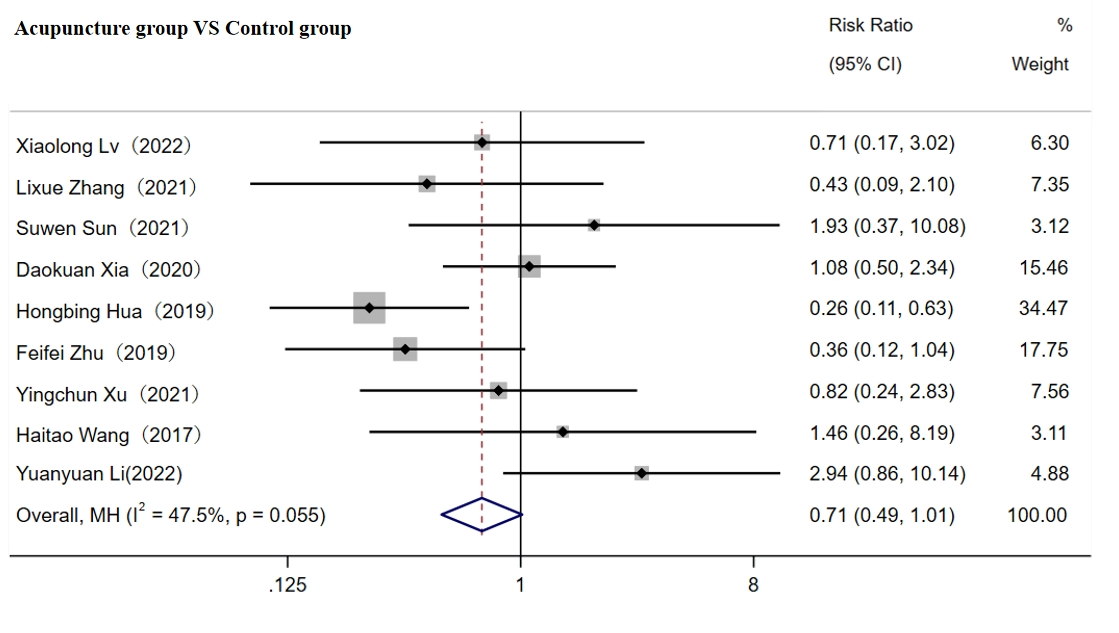


**Figure 12 Funnel plot of publication bias assess.(A: publication bias assess on TNF-α; B: publication bias assess on IL-6; C: publication bias assess on hs-CRP; D: publication bias assess on TCER; E: publication bias assess on NIHSS scores; F: publication bias assess on CRP; G: publication bias assess on IL-1β; H: publication bias assess on AE rate)**
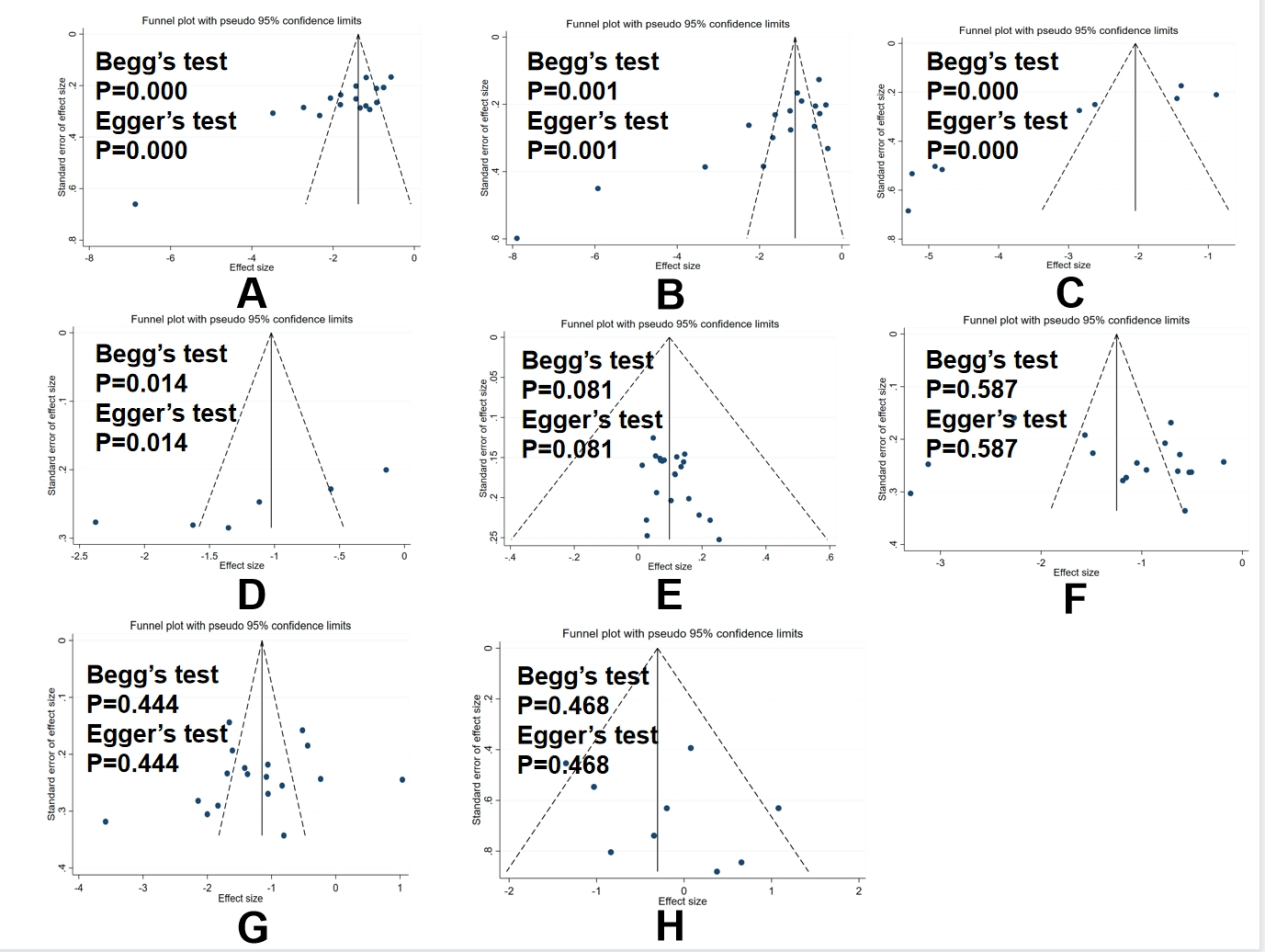


**Figure.13 Results of TSA（A:TNF-α, B: IL-6, C: hs-CRP, D: TCER, E: NIHSS scores, F: CRP, G: IL-1β, H: AE rate, RIS: Required Information Size）**

**
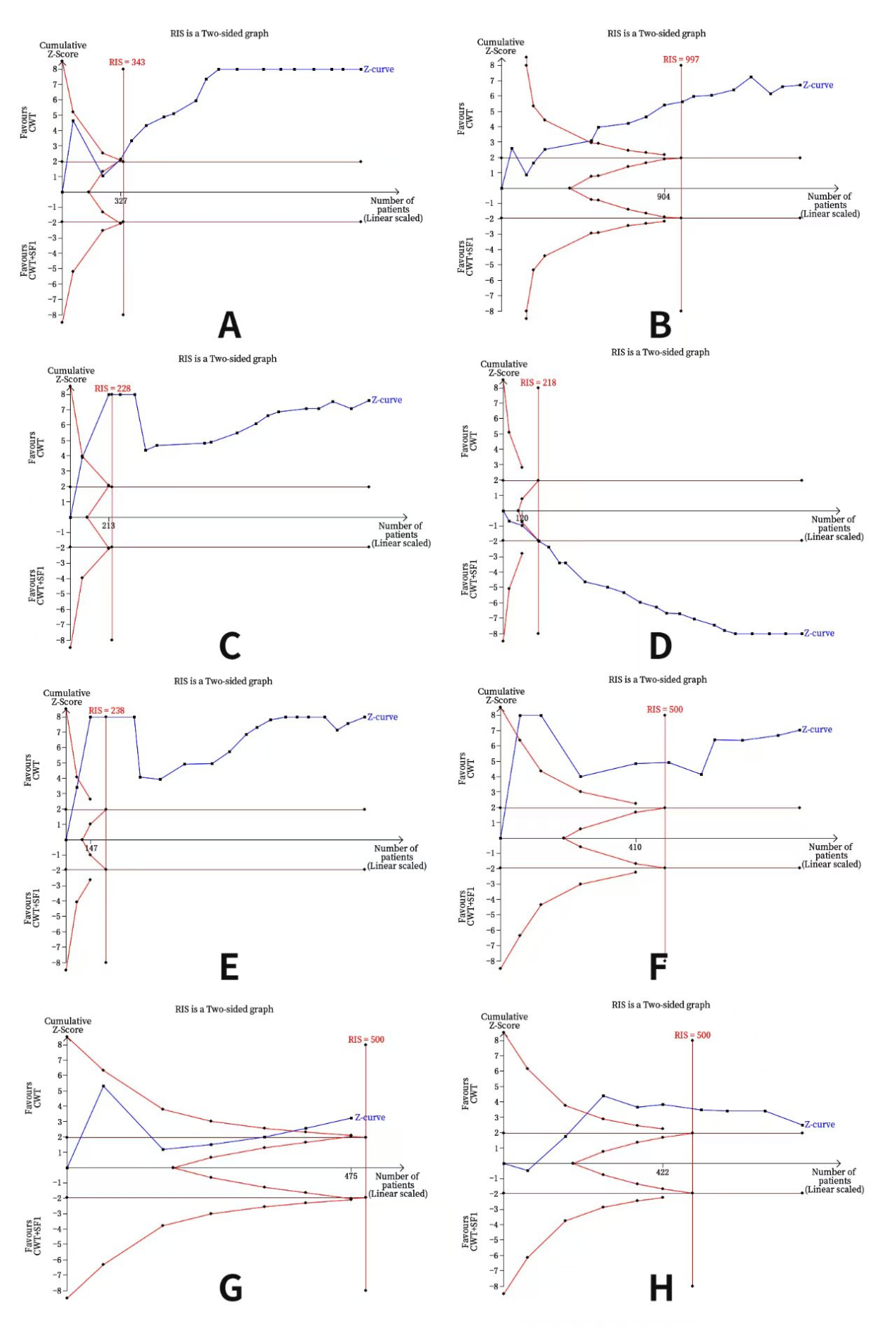
**
